# Exploring the Ambiguous Status of Coagulase-Negative Staphylococci in the Biosafety of Fermented Meats: The Case of Antibacterial Activity Versus Biogenic Amine Formation

**DOI:** 10.3390/microorganisms8020167

**Published:** 2020-01-24

**Authors:** David Van der Veken, Rafik Benhachemi, Christina Charmpi, Lore Ockerman, Marijke Poortmans, Emiel Van Reckem, Chris Michiels, Frédéric Leroy

**Affiliations:** 1 Research Group of Industrial Microbiology and Food Biotechnology (IMDO), Faculty of Sciences and Bio-engineering Sciences, Vrije Universiteit Brussel, 1050 Brussels, Belgium; David.Van.Der.Veken@vub.be (D.V.d.V.); Christina.Charmpi@vub.be (C.C.); Lore.Ockerman@vub.be (L.O.); Emiel.Van.Reckem@vub.be (E.V.R.); 2 Laboratory of Food Microbiology and Leuven Food Science and Nutrition Research Centre (LFoRCe), KU Leuven, B-3001 Leuven, Belgium; rafik.benhachemi@kuleuven.be (R.B.); marijke.poortmans@kuleuven.be (M.P.); chris.michiels@kuleuven.be (C.M.)

**Keywords:** *Staphylococcus*, *Clostridium botulinum*, antibacterial activity, biogenic amines, UPLC-MS/MS, fermented meat

## Abstract

A total of 332 staphylococcal strains, mainly isolated from meat, were screened for antibacterial activity. Eighteen strains exhibited antibacterial activity towards species within the same genus. These antibacterial strains were further screened against *Clostridium botulinum*, to assess their potential as anticlostridial starter cultures for the development of fermented meat products without added nitrate or nitrite. Only *Staphylococcus sciuri* IMDO-S72 had the ability to inhibit all clostridial strains tested, whilst displaying additional activity against *Bacillus cereus*, *Listeria monocytogenes* and *Staphylococcus aureus*. Apart from their potential as bioprotective cultures, the staphylococcal collection was also screened for biogenic amine production, as these compounds may compromise food quality. To this end, ultra-high-performance liquid chromatography coupled to tandem mass spectrometry (UPLC-MS/MS) was applied. A low incidence of biogenic amine production was found, with tyramine and β-phenylethylamine being the most prevalent ones. Concentrations remained relatively low (< 52 mg/L) after a prolonged incubation period, posing no or little threat towards food safety. Taken together, *S. sciuri* IMDO-S72 could serve as an interesting candidate for the bioprotection of fermented meats as it showed promising antibacterial activity as well as absence of biogenic amine production.

## 1. Introduction

The fermentation of meat is a preservation technique that has been practiced for millennia to cope with the perishable nature of this highly nutritious food matrix [1]. Notwithstanding the fact that fermented meats stood the test of time, they remain occasionally sensitive to the outgrowth of pathogens such as *Clostridium botulinum*, *Listeria monocytogenes*, and *Staphylococcus aureus*. On top of acidification, biosafety is reinforced by the application of salting, drying, and the use of food preservatives. Nitrite salts (and/or their nitrate precursors) are such preservatives that play a versatile role in the curing process of fermented meats. By formation of nitrosylmyoglobin, these preservatives are responsible for the stable red colour of cured meats. Nitrite also aids in establishing a cured meat flavour and the prevention of lipid oxidation [2]. Lastly, nitrite is well known for its antibacterial activity against pathogens such as *C. botulinum*, *Escherichia coli*, *L. monocytogenes*, *Salmonella enterica*, and *S. aureus* [3]. From the pathogens that occur in cured fermented meats, *C. botulinum* is among the most sensitive towards nitrite due to its limited potential to cope with oxidative/nitrosative stress [3]. Despite their functionalities in fermented meats, the addition of nitrate and nitrite salts has been susceptible to controversy. Formation of carcinogenic *N*-nitrosamines can occur when nitrite-derived nitric oxide reacts with biogenic amines or after ingestion [2,4,5]. Nowadays, nitrate and nitrite addition are well regulated (EU Regulation no. 1129/2011), limiting the concentration to 150 mg/kg for both nitrate and nitrite [6]. Consumer trends towards clean labels and the controversial status of nitrate and nitrite are nonetheless imposing a demand for removal of these compounds from the manufacturing process of fermented meat products [4,6]. This would however require an exhaustive assessment of the microbiological safety [7]. Although the incidence and outgrowth of *C. botulinum* in fermented meats is very low, countermeasures may be necessary to ensure the microbiological safety when decreasing or omitting nitrate and nitrite salts [6,7].

One of the possible solutions to counteract food pathogens in clean label foods, is the use of bioprotective starter cultures [8,9,10]. These cultures can inhibit spoilage and pathogenic bacteria by producing antimicrobial compounds, mostly bacteriocins [11,12]. Bioprotective cultures have mainly been obtained within the group of lactic acid bacteria (LAB) [13,14,15,16]. More specifically, the potential of antilisterial activity by food-grade LAB has already been assessed in several studies [17,18,19,20]. Besides LAB, constituting the major part of the natural microbial consortium in fermented meat, coagulase-negative staphylococci (CNS) could also serve as candidate bioprotective strains. Coagulase-negative staphylococci exhibit some key technological features, ensuring colour formation through nitrate/nitrite reductase as well as generating flavour through proteolytic and lipolytic activities [21]. Their antibacterial potential, however, remains poorly explored [10]. The fact that these bacteria are less studied may partly be due to their occasional association with opportunistic infections and certain safety hazards that can be encountered in some of the CNS species [22,23].

When screening for possible bioprotective strains, it is therefore of key importance that the presence of possible negative traits is also assessed. Safety hazards that are often linked with food-borne CNS are antibiotic resistance genes, enterotoxin production, and the presence of decarboxylases that can cause biogenic amine production [22]. Biogenic amines are typically linked with fermented foods high in protein content such as cheese, fish, and meat [24,25]. Vasoactive biogenic amines such as tyramine, β-phenylethylamine (PEA), and histamine can cause adverse effects in humans when consumed in too high levels. Other biogenic amines such as putrescine and cadaverine are less toxic and commonly associated with enterobacteria together with an inferior quality of the raw material [26,27]. Nevertheless, they can potentiate the toxicity of other biogenic amines, especially histamine, by competitive inhibition of the detoxifying enzymes [28]. Despite the general scientific consensus that such compounds need to be kept under certain levels to avoid food poisoning, European legislation only imposes a limit for histamine levels in fish products (EC No 2073/2005). In fermented meats, the most encountered biogenic amines are tyramine, putrescine, and cadaverine, whereas histamine is seldomly detected [26,29,30,31,32,33]. Tyramine is mostly associated with LAB such as *Carnobacterium* spp., *Enterococcus* spp., and *Lactobacillus curvatus*, whereas putrescine and cadaverine are linked with the presence of enterobacteria [26,27,29,30,31,33,34,35,36,37]. Coagulase-negative staphylococci have also been studied regarding their biogenic potential, albeit to a lesser extent. Generally, biogenic amine production appears to be less prevalent in CNS although *Staphylococcus carnosus* is often reported as a tyramine and PEA producer [22,23,29,32,38,39,40,41,42,43]. Irrespective of this low incidence, assessing the capability to produce biogenic amines remains an important aspect when screening for unconventional strains in view of an application within a food matrix, especially considering the strain-dependent character of this trait [29,44].

In view of bioprotective starter culture selection, the present study aimed at exploring the prevalence of antibacterial activity in a collection of staphylococci, that have been mainly isolated from meat, towards closely related species. In a next step, their potential to inhibit important food pathogens, especially *C. botulinum*, was analysed as to assess their potential for bioprotection. Finally, the study aimed at assessing whether biogenic amine formation can pose a problem within this bacterial group.

## 2. Materials and Methods

### 2.1. Staphylococcal Strains

A total of 332 staphylococcal strains were subjected to a screening to assess their antibacterial potential (Table S1 and Figure S1). The strain collection comprises 20 different species isolated from different ecological niches such as teat apex skin, milk, meat starter cultures, and raw and fermented meats, whereby the majority (86%) originated from a meat-related context. About one third of the collection (106 strains) was already available in the collection of the research group of Industrial Microbiology and Food Biotechnology (IMDO; Vrije Universiteit Brussel, Brussels, Belgium). To obtain a larger set of strains, additional isolations were performed from raw and fermented pork. Bacterial isolates were picked up from mannitol salt phenol-red agar (MSA; Merck, Darmstadt, Germany), which were then classified and identified based on their (GTG)_5_-PCR fingerprints of genomic DNA [45]. Identity was established by sequencing of the *rpoB* and/or *tuf* gene of the representative isolates, using the basic local alignment search tool (BLAST) and the Genbank database of the National Center for Biotechnological Information (http://www.ncbi.nih.gov/BLAST). All strains were stored in brain heart infusion (BHI) broth (Oxoid, Basingstoke, UK) containing 25% (v/v) glycerol at −80 °C.

### 2.2. Screening for Antibacterial Activity

A screening was performed according to a deferred antagonism test [46]. Staphylococcal strains were prestreaked on MSA, after which a single colony was picked up to inoculate 5 mL of BHI broth. After overnight incubation, 10 µL of the inoculated BHI was applied as a central streak (width of 5 mm) on BHI agar (1.5% *w*/*v*) and incubated for 24 h. Afterwards, the agar medium was flipped and overlayed with soft BHI agar (0.7% *w*/*v*) inoculated with 100 µL of an overnight grown indicator strain. All incubations were performed at 30 °C. Antibacterial activity was scored according to the width of the inhibition zone (IZ) measured from the central streak; 0 = no inhibition, 1 = IZ ≤ 1 mm, 2 = 1 mm ≤ IZ ≤ 4 mm, 3 = 4 mm ≤ IZ ≤ 8 mm, and 4 = IZ ≥ 8 mm. The complete set used in the screening can be found in Supplementary Table S2.

### 2.3. Screening for Anticlostridial Activity

Eighteen antibacterial staphylococcal strains were tested for antagonistic activity against a total of 33 *C. botulinum* strains, (both group I and group II; Supplementary Table S3), using a spot-on-lawn assay [47,48]. All manipulations of *C. botulinum* were carried out in an anaerobic workstation [Whitley DG250 (Don Whitley Scientific, Bingley, UK); 80% N_2_, 10% CO_2_, and 10% H_2_] at 30 °C. All media/solutions were deoxygenated prior to use by anaerobic incubation overnight. *Clostridium botulinum* strains were grown from spore suspensions on reinforced clostridial agar (RCA) [38 g/L of reinforced clostridial medium (RCM; Oxoid) and 15 g/L of bacteriological agar (Neogen, Lansing, MI, USA)] for 48 h, then single colonies from each strain were grown in RCM broth for 24 h and spread on RCA as indicator strain for the spot-on-lawn assays. Staphylococcal strains were grown on BHI agar (1.5% *w*/*v*) at 30 °C for 24 h, after which single colonies of each candidate strain were grown in BHI broth for 24 h to be used directly for cell-suspension (CS) spotting and heat-treated cell-free supernatant (HT-CFS) preparation (centrifugation at 21,420× *g*, for 15 min at 4 °C followed by a heat treatment at 60 °C for 15 min). Five µL of (i) CS and (ii) HT-CFS were spotted against 9 *C. botulinum* group I and 24 *C. botulinum* group II strains. Sensitivity of indicator strains was assessed after 24 h of incubation at 30 °C under anaerobic conditions, by the observation of inhibition halos around staphylococcal CS and HT-CFS spots.

### 2.4. Assessment of Temperature and Proteinase K on the Stability of Antibacterial Activity in Broth from Staphylococcus sciuri IMDO-S72 and its Spectrum of Activity

The spot-on-lawn assay was carried out with *Staphylococcus sciuri* IMDO-S72 against six Gram-positive (*Bacillus cereus*, *Bacillus subtilis*, *Lactococcus lactis*, *Lactobacillus sakei*, *L. monocytogenes* and *S. aureus*) and two Gram-negative (*E. coli* and *S. enterica* Typhimurium) bacteria. Indicator strains were prepared following the same steps as described earlier. *Bacillus cereus* and *L. monocytogenes* were grown for 24 h and *L. lactis*, *L. sakei* and *S. aureus* for 48 h on BHI agar (1.5% *w*/*v*) at 30 °C, followed by 24 h of incubation in BHI broth. *Bacillus subtilis*, *E. coli*, and *S. enterica* Typhimurium were grown on LB agar [10 g/L Trypton (Neogen), 5 g/L yeast extract (Oxoid), 5 g/L NaCl (Fisher Chemical, Hampton, NH, USA), 15 g/L bacteriological agar (Neogen)] for 24 h, followed by incubation in liquid LB for 24 h at 37 °C. Indicator strains were exposed to spotting as described before. Sensitivity was assessed after 24 h of incubation under aerobic conditions at 30 °C and 37 °C for BHI and LB agar, respectively. To assess protease sensitivity and thermal stability, HT-CFS was subject to treatment with proteinase K [~2 mg/ml (Thermo Scientific, Waltham, MA, USA)] at 56 °C for 2 h and to additional heat treatments at 60, 80, and 90 °C for 20 min and at 100 °C for 10 min, before use in a spot-on-lawn assay.

### 2.5. Analysis of Biogenic Amines

#### 2.5.1. Growth Conditions and Sampling

All 332 staphylococcal strains were first streaked on MSA and incubated at 30 °C. After sufficient growth, one colony per strain was picked up and transferred to 10 mL BHI broth serving as a nutrient-rich medium and incubated at 30 °C for two weeks. A sample taken from the uninoculated BHI broth was used as starting point, to take into account initial background concentrations of biogenic amines already present. Samples were taken after 7 and 14 d of incubation and were immediately processed. Cell-free supernatant was obtained via centrifugation (5000× *g* for 20 min at 4 °C). Samples were deproteinized by mixing 250 µL of cell-free supernatant with 250 µL of acetonitrile containing 0.15% heptafluorobutyric acid (HFBA, ≥99%; Sigma-Aldrich, St. louis, MO, USA). Afterwards, samples were centrifuged (13,000× *g* for 15 min at 4 °C) and filtered over H-PTFE filters (Merck) into microvials before injection into the column (2 µL).

#### 2.5.2. Determination of Biogenic Amines by Ultra-High-Performance Liquid Chromatography Coupled to Tandem Mass Spectrometry (UPLC-MS/MS)

The concentrations of agmatine, cadaverine, histamine, β-phenylethylamine, putrescine, tryptamine, tyramine, spermidine and spermine were measured by UPLC-MS/MS. The analysis was performed with an Acquity UPLC system, equipped with an HSS T3 column (length: 150 mm; inner diameter: 2.1 mm; particle size: 1.8 µm; pore size: 100 Å) and a TQ tandem mass spectrometer with a ZSpray™ electrospray ionization source used in positive ionization mode (Waters, Milford, MA, USA).

The mobile phase consisted of an ultrapure water-acetonitrile mixture (95:5, v/v) with 0.1% (v/v) of HFBA as an ion-pairing reagent (eluent A) and an ultrapure water-acetonitrile mixture (5:95, v/v) with 0.1% (v/v) HFBA (eluent B). The following gradient (0.23 mL/min) was applied: 0.0–5.0 min, isocratic 95% A and 5% B; 5.0–7.0 min, linear from 95% to 80% A and from 5% to 20% B; 7.0–8.0 min, linear from 80% to 30% A and from 20% to 70% B; 8.0–11.0 min, isocratic 30% A and 70% B; 11.0–12.0 min, linear from 30% to 95% A and from 70% to 5% B; 12.0–15.0 min, isocratic 95% A and 5% B.

The selected reaction monitoring (SRM) method, including cone voltage (V) and collision energy (eV), was optimized via IntelliStart (Waters), and the dwell time was adjusted to ensure a minimum of 10 scans for each compound peak. The following mass spectrometric settings were used: capillary voltage, 3.50 kV; source temperature, 150 °C; desolvation temperature, 450 °C; cone gas flow, 0 l/h; desolvation gas flow, 800 l/h. Cone voltage and collision energy were dependent on the compound detected and are given in Table 1. Quantification of the targeted biogenic amines was achieved through external calibration in triplicate. The preparation of the standard solutions was equivalent to that of the samples as described earlier. All biogenic amines were obtained from Sigma-Aldrich and had a purity level of 98% or higher except for agmatine and spermine which had a purity of 97% or higher and 96% or higher, respectively.

### 2.6. Graphical Representation

Heatmaps and metabolite profiles were constructed using the package ComplexHeatmap [49] and ggplot2 [50] in Rstudio [51].

## 3. Results

### 3.1. Prevalence of Antibacterial Activity in Staphylococci

**Figure 1:**
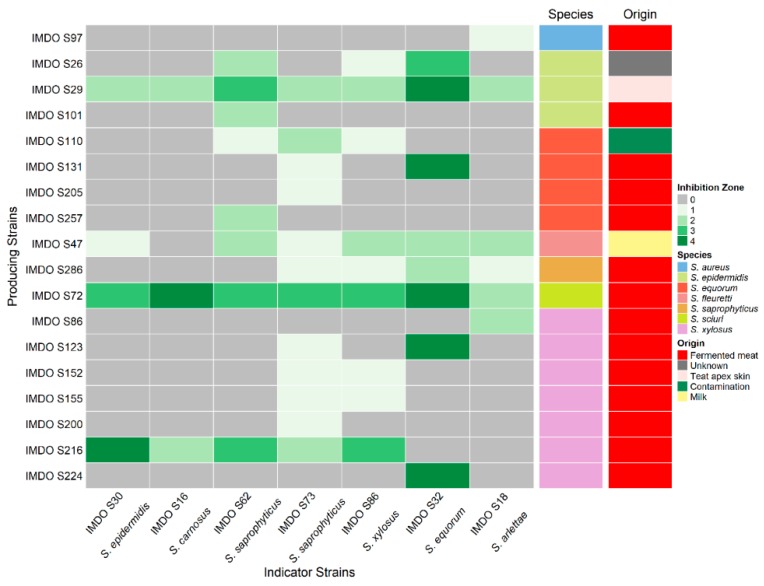
Heatmap of activity spectra of antibacterial *Staphylococcus* strains against seven indicator strains. The indicator strains used were isolated from different origins; IMDO-S30, fermented meat; IMDO-S16, fermented meat; IMDO-S62, teat apex skin; IMDO-S73, milk; IMDO-S86, fermented meat; IMDO-S32, teat apex skin; and IMDO-S18, teat apex skin. On the right side, species and origin of the producing strains are represented. Producing strains are ordered alphabetically according to species name.

Within the *Staphyloccocus* collection tested, a relatively low prevalence of antibacterial production was found as only 18 of the 332 strains (5%) exhibited activity towards one or more indicator strains (Figure 1). A high variability was encountered in the inhibition spectra towards the indicator strains, comprising both very narrow as well as broad activity spectra. Antibacterial activity was most common for *Staphylococcus xylosus, Staphylococcus equorum,* and *Staphylococcus epidermidis* representing 78% of the antibacterial strains. However, one has to bear in mind that these species were also abundantly present in the collection, which may create an overrepresentation. Fourteen inhibiting strains originated from fermented meat, while it is notable that no antibacterial strains from raw meat were found. Next to the low prevalence, antibacterial production was a very strain-dependent trait.

Antibacterial sensitivity was also strongly strain-dependent, as can be seen from the different inhibition patterns of *Staphylococcus saprophyticus* IMDO-S62 and IMDO-S73. From the minority of antibacterial strains, only *S. epidermidis* IMDO-S29 and *S. sciuri* IMDO-S72 were able to inhibit all of the indicator strains, which makes them promising candidates for screening of genes and isolation of the corresponding antibacterial compounds that may be exploited for food safety purposes.

### 3.2. Antibacterial Activity by Staphylococcus sciuri IMDO-S72

Strains that displayed antibacterial activity against one or more indicator strain were further investigated as to their potential to inhibit *C. botulinum*. A set of non-antibacterial staphylococcal strains was also included, comprising most species that had members with antibacterial activity, to serve as a negative control and to assess the possibility of missing anticlostridial activity in such strains. After 24 h of incubation all *C. botulinum* group I and II strains tested, were able to grow in the presence of CS and HT-CFS for 17 out of the 18 antibacterial staphylococcal isolates tested. Among the 18 isolates identified as antagonistic to other staphylococci, *S. sciuri* IMDO-S72 exhibited antagonism against all *C. botulinum* strains without group specificity. The inhibition zones ranged from 2 to 5 mm. Apart from the CS, treatment with HT-CFS from *S. sciuri* IMDO-S72 resulted in growth inhibition, suggesting the extracellular release of one or several antibacterial compounds that can inhibit the growth of *C. botulinum* vegetative cells. None of the strains included in the negative set showed activity towards *C. botulinum*.

To get a further view on the nature of the antibacterial compound(s) of *S. sciuri* IMDO-S72, the spot-on-lawn experiment was extended to other foodborne pathogens (Table S4). *Staphylococcus sciuri* IMDO-S72 did not show antagonism towards the Gram-negative bacteria *E. coli* and *S. enterica* Typhimurium while it inhibited ten out of eleven Gram-positive bacteria. *Bacillus subtilis*, *L. lactis*, *L. monocytogenes*, *L. sakei* and *S. aureus* and remarkably five out of six *B. cereus* strains were inhibited. *Bacillus cereus* ATCC 14579 growth was not antagonized by both CS and HT-CFS of *S. sciuri* IMDO-S72. It could be hypothesized that *S. sciuri* IMDO-S72 produces a similar antibacterial compound to that of *B. cereus* ATCC 14579, explaining the absence of an antagonistic effect towards this strain. Further identification of the antibacterial activity of *S. sciuri* IMDO-S72 is however imperative to confirm this statement. Apart from its rather wide activity towards Gram-positive bacteria, the HT-CFS was also subjected to multiple heat treatments as well as to proteinase K. It turned out that the antagonism was not impacted by the applied heat treatments, reaching 20 min at 90 °C and 10 min at 100 °C, indicating a rather heat-stable antibacterial compound or compounds. Also, the HT-CFS samples were still active after six months of storage at 4 °C. Proteinase K treatment did not affect the antibacterial activity of HT-CFS from *S. sciuri* IMDO-S72, which might suggest a non-proteinaceous nature.

### 3.3. Low and Strain-Dependent Biogenic Amine Production in Staphylococci, Marked by Simultaneous Production of Tyramine and β-Phenylethylamine

**Figure 2:**
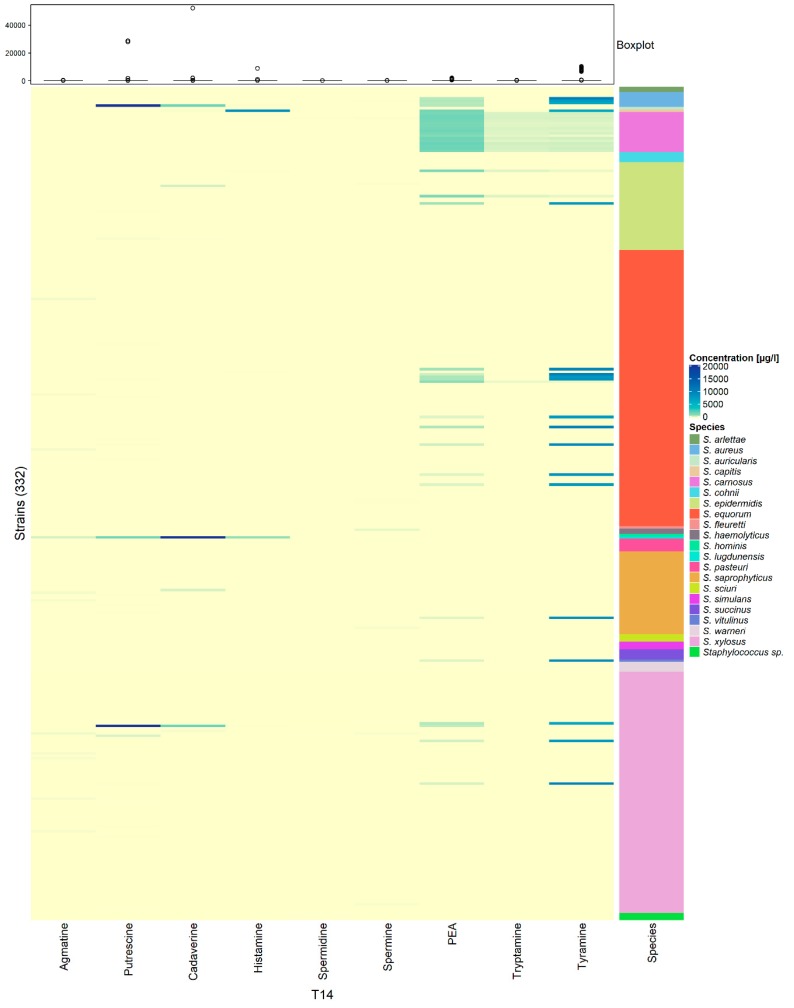
Heatmap showing the increase in different biogenic amine concentrations (µg/L) after 14 d of incubation in BHI broth, for all 332 staphylococcal strains (ordered alphabetically according to species name). A concentration range from 0 to 20,000 µg/L was chosen to display most of the measured samples, while the indicative boxplot shows the complete concentration range per biogenic amine for all strains.

**Figure 3:**
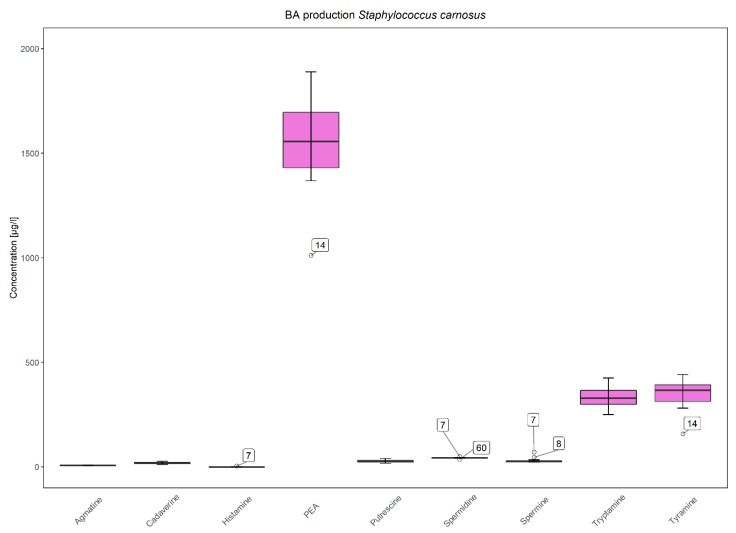
Average increase in biogenic amines (in µg/L) for all *Staphylococcus carnosus* strains (16) after 14 d of incubation in BHI broth. Outliers are indicated with the corresponding strain number (being *S. carnosus* IMDO-S7, IMDO-S8, IMDO-S14, and IMDO-S60).

The concentrations of biogenic amines in BHI were initially very low being 11.07, 26.21, 33.68, 34.16, and 37.92 µg/L for agmatine, cadaverine, putrescine, spermidine and spermine, respectively. Histamine, PEA, tryptamine, and tyramine were under the limit of quantification (LOQ) and assumed zero. The limits of quantification were 11.01, 11.14, 11.56, 16.59, 8.32, 6.64, 8.67, 16.13 and 16.06 µg/L for agmatine, cadaverine, histamine, PEA, putrescine, tryptamine, tyramine, spermidine and spermine, respectively. After 14 d of incubation, tyramine and PEA were the most frequently produced biogenic amines (Figure 2). Their production occurred always simultaneously except for two strains namely *S. aureus* IMDO-S127 and *S. xylosus* IMDO-S128. Mostly, tyramine was produced in higher amounts compared to PEA, except for *S. epidermidis* IMDO-S26 and IMDO-S101 as well as for *S. equorum* IMDO-S192. For *S. carnosus* strains, the opposite ratio was true, although less distinct, where PEA was produced in higher amounts relative to tyramine (Figure 3). Although the production of biogenic amines was generally a strain-specific trait, such specificity was not found for *S. carnosus* as this species consistently produced PEA, tyramine, as well as tryptamine.

The incidence of histamine production was very low, as only two strains were able to produce this compound in substantial amounts. This was the case for *Staphylococcus capitis* IMDO-S186 and *Staphylococcus lugdunensis* IMDO-S92, reaching 8763.09 µg/L and 903.26 µg/L, respectively. *Staphylococcus lugdunensis* IMDO-S92, apart from its histamine production, also produced agmatine, cadaverine, and putrescine, whereby cadaverine reached a concentration of around 52 mg/L. This was the highest measured concentration compared to all other staphylococcal isolates. Generally, however, the occurence of putrescine and cadaverine was relatively low, both also being produced together by *S. aureus* IMDO-S127 and *S. xylosus* IMDO-S128. In contrast, *S. epidermidis* IMDO-S93 and *S. saprophyticus* IMDO-S115 only produced cadaverine, *S. xylosus* IMDO-S129 was a putrescine producer. Apart from some small sporadic increases, the concentration of agmatine, spermidine, and spermine remained more or less unchanged.

**Figure 4:**
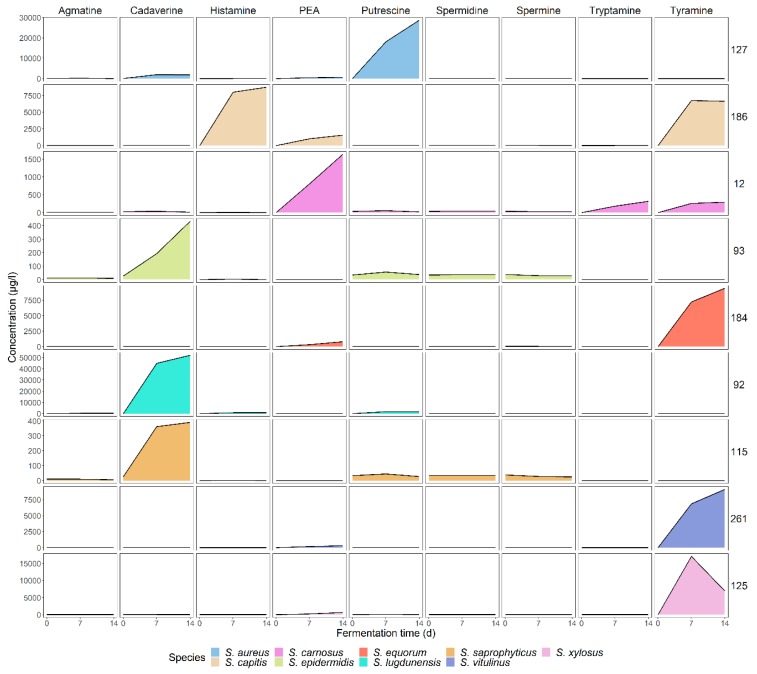
Biogenic amine profiles of fermentations in BHI broth at 30 °C during 14 d. Only selected strains are shown, namely *S. aureus* IMDO-S127, *S. capitis* IMDO-S186, *S. carnosus* IMDO-S12, *S. epidermidis* IMDO-S93, *S. equorum* IMDO-S184, *S. lugdunensis* IMDO-S92, *S. saprophyticus* IMDO-S115, *S. vitulinus* IMDO-S261, and *S. xylosus* IMDO-S125. Scaling for each depicted strain was adapted to improve the readability of the corresponding profiles.

Since the production process of fermented meats can take several weeks, it is most likely that even after acidification and drying the microbiota remains metabolically active. To evaluate if longer fermentation/incubation times would lead to higher concentrations of biogenic amines, samples were taken and analyzed after 7 and 14 d. Profiles of the most notable biogenic amine producing strains are shown in Figure 4. Aside from the difference in concentrations, the profile of *S. aureus* IMDO-S127, producing putrescine and to a lesser extent cadaverine, was also shared by *S. xylosus* IMDO-S128. Although cadaverine ceased to increase after 7 d, putrescine kept increasing until the end of the fermentation. The profile of *S. capitis* IMDO-S186 was unique, as it was the only strain that produced histamine besides tyramine and PEA. A more pronounced levelling-off was observed here for histamine and tyramine after 7 d, but not for PEA. The profile of *S. carnosus* IMDO-S12 is representative for all *S. carnosus* strains analyzed, as well as for *S. epidermidis* IMDO-S26, IMDO-S101, and *S. equorum* IMDO-S192. Also, here, a longer fermentation time gave rise to higher amounts of PEA and tryptamine, as they increased almost linearly across the incubation period. *Staphylococcus epidermidis* IMDO-S93 only produced cadaverine, and although the concentration remained relatively low, the most marked increase was found between 7 and 14 d. The profile of *S. equorum* IMDO-S184, marked by a high tyramine production relative to PEA, was representative for all aminogenic *S. equorum* strains, except IMDO-S192 as mentioned above, as well as for *S. epidermidis* IMDO-S153, *S. saprophyticus* IMDO-S276, *Staphylococcus vitulinus* IMDO-S261, and *S. xylosus* IMDO-S132 and IMDO-S179. A further increase after 7 d was also found here for both tyramine and PEA. *Staphylococcus lugdunensis* IMDO-S92 produced the highest number of biogenic amines, more specific cadaverine, but also produced agmatine, histamine, and putrescine (although their increase is masked due to the high concentration of cadaverine). The profile of *S. saprophyticus* IMDO-S115 was similar to that of *S. epidermidis* IMDO-S93, as mentioned above, but the increase after 7 d was less pronounced. In the profile of *S. xylosus* IMDO-S125, a decrease was encountered for tyramine after 7 d which was also found for *S. aureus* IMDO-S124 and IMDO-S126. These were the only strains for which a decrease was observed after production of the corresponding biogenic amine, which suggest an ability to metabolise these compounds.

Longer fermentation times can give rise to higher concentrations of biogenic amines, as was seen for some strains. Leaving out the negligible increases in agmatine and spermine, the incidence of biogenic amine production was overall relatively low in staphylococci, with only 14% of the strains exhibiting production. The complete dataset, containing measured concentrations for the total staphylococcal library after 7 and 14 d, can be found in Supplementary Table S5.

## 4. Discussion

The most abundant species within the staphylococcal library were *S. equorum*, *S. xylosus*, *S. epidermidis*, *S. saprophyticus,* and *S. carnosus*. As this study focused on meat-born staphylococci, the preponderation of these species can easily be linked to their relatedness with fermented meat. Indeed, *S. equorum*, *S. xylosus,* and *S. saprophyticus* are often isolated from spontaneously fermented sausages [52]. Also, *S. epidermidis* has been frequently isolated from fermented meat, being a part of the common subdominant microbiota but can sometimes increase in abundance under mild pH and higher fermentation temperatures [53,54]. As for *S. carnosus*, this species is seldomly isolated from spontaneously fermented meats, which provides a striking contrast to its common use as meat starter culture [55].

Despite their beneficial role in food matrices, in particular fermented meat, fish, and some cheeses, the status of CNS remains ambiguous as these bacteria are often linked with human infections. Most of them appear as harmless commensals on mammal skin, but some species can act as opportunistic pathogens. *Staphylococcus epidermidis*, a common colonizer of human skin, is the most frequent cause of nosocomial infections and colonization of indwelling medical devices, whereas *S. saprophyticus* is often involved in urinary tract infections [56,57]. Although some CNS are already used as starter cultures for food fermentation, their ambivalent status may be the reason why they are less explored regarding their antibacterial potential for food preservation compared to LAB which are often granted GRAS (generally recognised as safe) or QPS (qualified presumption of safety) status. Because the frequency of occurrence and the activity spectra of antibacterial activity within the group of CNS are still relatively underexplored, an extensive screening was performed in the present study. It turned out that the general prevalence of antibacterial activity was rather low, with a frequency of merely 5%. Moreover, presence of antibacterial activity was strain- rather than species-dependent, which can be ascribed to the fact that such compounds are often encoded on mobile genetic elements that can be exchanged by horizontal gene transfer between and even across species [58,59,60,61,62].

In other studies that focused on food-borne staphylococci, varying concentrations of antibacterial activity were reported ranging from 8% to 51% [63,64,65,66,67]. If the fraction of coagulase-positive staphylococci is ignored, antibacterial incidence drops to 4–17%, indicating a rather low dissemination in CNS isolated from food systems, which corresponds with the 5% level of the present study [63,64,65,66,67]. Other screenings that focused on staphylococci from ecological niches other than food, including teat apex skin, human skin and nasal cavities reported varying frequencies of antibacterial activity ranging from 6% to 84% [58,68,69,70]. Especially staphylococci isolated from human body surfaces were reported to display a high frequency of bacteriocin production [58,70]. Such antibacterial traits can indeed confer extra competitiveness to the producing strains, canceling out other competitors within the same ecological niche. It can be expected that competitive exclusion based on such a trait is more paramount in stressing and nutrient-poor environments compared to nutrient-rich niches [11,58]. Since most screening experiments focused on phenotypic expression of antibacterial activity, the genetic potential may be underestimated, as some antagonistic compounds might only be produced under specific conditions [65,68]. For bacteriocins, for instance, quorum-sensing systems may be in place so that in some cases a certain cell-density threshold has to be exceeded for production to occur [71,72,73]. Also, specific conditions within a food matrix, as well as the effects of temperature and pH, may result in stimulation or loss of bacteriocin activity [10,74].

The strains that showed antibacterial activity towards closely related indicator strains were further assessed to evaluate their potential to inhibit *C. botulinum*. The use of anticlostridial CNS strains could help to compensate for the removal of nitrate and nitrite in clean-label fermented meats. The incidence of *C. botulinum* in meat is in the range of 0% to 73%, depending on the meat type [7]. Antibacterial CNS strains were tested against group I and group II *C. botulinum* strains isolated from different sources. The focus on these groups is related to the fact that they can produce toxins A, B, E, and F that are responsible for almost all cases of botulism in humans [7].

Only *S. sciuri* IMDO-S72 was able to inhibit *C. botulinum,* together with other food pathogens such as *B. cereus*, *L. monocytogenes*, and *S. aureus*. This strain can thus not only be promising as an antibotulinal agent but also in view of a broader food safety impact. Yet, it needs still to be further determined if this strain can sufficiently proliferate in a meat matrix while expressing its antibacterial activity, as bacterial behaviour can be profoundly affected by the processing environment, such as the presence of specific substrates or the effect of pH and temperature conditions [74,75]. The fact that LAB were also inhibited, for instance, can hamper its use as a bioprotective starter culture in meat fermentations as its antibacterial activity can thereby postpone or even halt the normal acidification process [76]. Also, the nature and identity of the antibacterial compound(s) will have to be elucidated, which could be either bacteriocin-like peptides or non-ribosomally synthesized, non-proteinaceous compounds, such as surfactin and daptomycin [77,78,79]. The antibacterial activity of such compounds mostly resides in their interaction with the cytoplasmic membrane, compromising membrane permeability and integrity, while producing strains protect themselves by specific adaptations in cell wall and cell membrane homeostasis or by dedicated efflux pomps [77,78,79,80].

The inclusion of a negative set in the screening against *C. botulinum* strains did not give any positive results. If absence of activity against closely related strains is found, it can be assumed that it is rather unlikely that a more distinct species will be inhibited. Of all bacteriogenic strains, *S. sciuri* IMDO-S72 indeed exhibited the most intense antagonistic activity towards other *Staphylococcus* species, indicating the presence of a rather broad-spectrum compound or compounds (as confirmed when testing this strain against other pathogenic bacteria). Nevertheless, it cannot be ruled out that strains with anticlostridial activity were missed based on this experimental set-up.

However, apart from their antibacterial potential, CNS might also possess negative traits that can hinder their use in food such as the presence of antibiotic resistance genes, phages, virulence factors and biogenic amines [81,82]. The production of the latter appeared to be a very strain-dependent trait during the present study. Again, the genetic location of such genes on unstable plasmids is often referred to as an explanation for this strain-dependency of phenotypes [25,44,83,84]. *Staphylococcus carnosus*, however, is an exception in this case as it appears to be a consistent producer of PEA, tryptamine, and tyramine. This species is known for its acid tolerance, which can be linked with the presence of arginine deiminase and decarboxylating activities of amino acids [43,85]. Decarboxylase activity has indeed already been linked with *S. carnosus* in previous studies, especially for PEA and tyramine [27,32,38,41,43].

Overall, the incidence of biogenic amine production remained relatively low within the staphylococcal collection analysed. Concentrations also remained relatively low, the highest being 52 mg/L of cadaverine, posing no direct threat for food safety even after an incubation time of 14 d. Noteworthy was the simultaneous production of tyramine and PEA, being the most prevalent biogenic amines produced by staphylococci. The dual activity of tyrosine-decarboxylase (TDC), acting on the structurally related amino acids tyrosine and L-phenylalanine, has already been reported in multiple studies [36,86,87,88,89]. Although these studies focused on LAB, it seems that the same unspecific activity of TDC towards L-phenylalanine can also be found for *Staphylococcus* species. In most cases, PEA is indeed produced in lower amounts in tyramine-producing strains, which might indicate the higher affinity of TDC towards tyrosine [24,88]. However, also here, *S. carnosus* can be distinguished from other staphylococcal species, as PEA is consistently produced in higher amounts compared to tyramine. Histamine production is generally reported to be uncommon in meat, especially when compared to fish products [30,32,90]. Only *S. capitis* IMDO-S186 and *S. lugdunensis* IMDO-S92 were able to substantially produce this biogenic amine. A histidine decarboxylase has indeed been identified in *S. capitis* and, although not widely encountered in meat, this species has been linked with histamine production before [38,39,91]. *Staphylococcus lugdunensis* has been mainly associated with the production of putrescine and cadaverine [38,39].

In general, *Staphylococcus* species have been reported to be poor biogenic amine producers, whereas the presence of these compounds is mostly associated with certain LAB and enterobacteria [22,23,26,29,30,31,32,33,34,36,37,38,39,40,42,43,92,93,94]. However, it cannot be excluded that specific conditions in fermented foods can induce higher decarboxylating activities. Anaerobicity and a low pH, which are conditions generally encountered in fermented meats, have been shown to favour the production of biogenic amines [24,26,95]. As shown in this study, longer fermentation times can allow further increase in biogenic amine concentrations. This can be linked to bacterial cells that remain metabolically active or, in addition, the release of decarboxylases due to cell lysis can also be responsible for biogenic amine accumulation as they can remain active even in the absence of viable cells [27,96]. Even when the level of biogenic amine concentrations was of no concern in this study, this effect should be taken into account for fermented meats, and food products in general, as this can compromise the shelf life and food safety of such products when applying novel starter cultures.

## 5. Conclusions

Coagulase-negative staphylococci can display both desirable and undesirable metabolic traits. Screening for antibacterial activity showed that the incidence of such beneficial phenotype is rather low and highly strain-dependent. Only *S. sciuri* IMDO-S72 exhibited anticlostridial activity together with activity towards other food-associated pathogens. Similarly, the occurrence of biogenic amine production was low overall and characterized by a strain-dependent character. Tyramine and PEA were the most prevalent amines, marked by a simultaneous production, whereas putrescine, cadaverine, and histamine were only rarely detected. As an antibacterial strain that does not lead to biogenic amine production, *S. sciuri* IMDO-S72 is an interesting candidate to be further explored as a bioprotective starter culture for meat fermentation. Nevertheless, a further characterization of this strain is necessary to elucidate the exact nature of the antibacterial activity as well as to confirm the absence of antibiotic resistance genes, phages, and virulence factors in view of a possible application in the food chain.

## Figures and Tables

**Table 1:** Selected reaction monitoring (SRM) transition (m/z), cone voltage (CV), and collision energy (CE) for the organic compounds measured by ultra-high-performance liquid chromatography coupled to tandem mass spectrometry (UPLC-MS/MS).

Organic Compound	Ionisation Mode	SRM	
		Transition (m/z)	CV (V)/CE (eV)
Agmatine	ES +	131.00 > 72.02	15/15
Cadaverine	ES +	103.08 > 86.04	15/12
Histamine	ES +	112.06 > 95.02	18/15
β-Phenylethylamine	ES +	122.08 > 105.02	23/12
Putrescine	ES +	89.05 > 72.02	11/11
Tryptamine	ES +	161.13 > 144.03	10/11
Tyramine	ES +	138.09 > 121.04	10/12
Spermidine	ES +	146.00 > 72.05	20/12
Spermine	ES +	203.22 > 129.12	22/12

## Supplementary Materials

The following are available online at https://www.mdpi.com/2076-2607/8/2/167/s1, Figure S1: Species diversity of the staphylococcal strain collection (332 strains).Table S1: Overview of the staphylococcal library used in this study. Table S2: Scores of antibacterial activities of the staphylococcal library towards seven staphylococcal indicator strains. Table S3: List of non-toxigenic *Clostridium botulinum* group I and group II used as indicator strains in spot-on-lawn assay. Table S4: Spot-on-lawn assay testing the antibacterial impact of *Staphylococcus sciuri* IMDO-S72 on different indicator strains. Table S5: Biogenic amine concentrations of the staphylococcal library after 7 and 14 d of incubation in BHI measured using UPLC-MS/MS.

## References

[1] Leroy F., Geyzen A., Janssens M., De Vuyst L., Scholliers P. (2013). Meat fermentation at the crossroads of innovation and tradition: A historical outlook. Trends Food Sci. Technol..

[2] De Mey E., De Klerck K., De Maere H., Dewulf L., Derdelinckx G., Peeters M.-C., Fraeye I., Vander Heyden Y., Paelinck H. (2014). The occurrence of N-nitrosamines, residual nitrite and biogenic amines in commercial dry fermented sausages and evaluation of their occasional relation. Meat Sci..

[3] Majou D., Christieans S. (2018). Mechanisms of the bactericidal effects of nitrate and nitrite in cured meats. Meat Sci..

[4] Sebranek J.G., Bacus J.N. (2007). Cured meat products without direct addition of nitrate or nitrite: What are the issues?. Meat Sci..

[5] Honikel K.-O. (2008). The use and control of nitrate and nitrite for the processing of meat products. Meat Sci..

[6] Hospital X.F., Hierro E., Stringer S., Fernández M. (2016). A study on the toxigenesis by *Clostridium botulinum* in nitrate and nitrite-reduced dry fermented sausages. Int. J. Food Microbiol..

[7] Lund B.M., Peck M.W., Labbé R.G., Garcia S. (2013). Clostridium botulinum. Guide to Foodborne Pathogens.

[8] Ravyts F., Barbuti S., Frustoli M.A., Parolari G., Saccani G., De Vuyst L., Leroy F. (2008). Competitiveness and antibacterial potential of bacteriocin-producing starter cultures in different types of fermented sausages. J. Food Protect..

[9] Di Gioia D., Mazzola G., Nikodinoska I., Aloisio I., Langerholc T., Rossi M., Raimondi S., Melero B., Rovira J. (2016). Lactic acid bacteria as protective cultures in fermented pork meat to prevent *Clostridium* spp. growth. Int. J. Food Microbiol..

[10] Sánchez Mainar M., Xhaferi R., Samapundo S., Devlieghere F., Leroy F. (2016). Opportunities and limitations for the production of safe fermented meats without nitrate and nitrite using an antibacterial *Staphylococcus sciuri* starter culture. Food Control.

[11] Cotter P.D., Hill C., Ross R.P. (2005). Bacteriocins: Developing innate immunity for food. Nat. Rev. Microbiol..

[12] Cotter P.D., Ross R.P., Hill C. (2013). Bacteriocins—A viable alternative to antibiotics?. Nat. Rev. Microbiol..

[13] Hugas M. (1998). Bacteriocinogenic lactic acid bacteria for the biopreservation of meat and meat products. Meat Sci..

[14] Ammor S., Tauveron G., Dufour E., Chevallier I. (2006). Antibacterial activity of lactic acid bacteria against spoilage and pathogenic bacteria isolated from the same meat small-scale facility: 1—Screening and characterization of the antibacterial compounds. Food Control.

[15] Hanlin M.B., Kalchayanand N., Ray P., Ray B. (1993). Bacteriocins of lactic acid bacteria in combination have greater antibacterial activity. J. Food Protect..

[16] Corsetti A., Gobbetti M., Smacchi E. (1996). Antibacterial activity of sourdough lactic acid bacteria: Isolation of a bacteriocin-like inhibitory substance from *Lactobacillus sanfrancisco* C57. Food Microbiol..

[17] Ghrairi T., Manai M., Berjeaud J.M., Frère J. (2004). Antilisterial activity of lactic acid bacteria isolated from rigouta, a traditional Tunisian cheese. J. Appl. Microbiol..

[18] Alves V.F., Martinez R.C.R., Lavrador M.A.S., De Martinis E.C.P. (2006). Antilisterial activity of lactic acid bacteria inoculated on cooked ham. Meat Sci..

[19] Albano H., Oliveira M., Aroso R., Cubero N., Hogg T., Teixeira P. (2007). Antilisterial activity of lactic acid bacteria isolated from “Alheiras” (traditional Portuguese fermented sausages): In situ assays. Meat Sci..

[20] Fontana C., Cocconcelli P.S., Vignolo G., Saavedra L. (2015). Occurrence of antilisterial structural bacteriocins genes in meat borne lactic acid bacteria. Food Control.

[21] Sánchez Mainar M., Stavropoulou D.A., Leroy F. (2017). Exploring the metabolic heterogeneity of coagulase-negative staphylococci to improve the quality and safety of fermented meats: A review. Int. J. Food Microbiol..

[22] Even S., Leroy S., Charlier C., Ben Zakour N., Chacornac J.-P., Lebert I., Jamet E., Desmonts M.-H., Coton E., Pochet S. (2010). Low occurrence of safety hazards in coagulase-negative staphylococci isolated from fermented foodstuffs. Int. J. Food Microbiol..

[23] Ruaro A., Andrighetto C., Torriani S., Lombardi A. (2013). Biodiversity and characterization of indigenous coagulase-negative staphylococci isolated from raw milk and cheese of North Italy. Food Microbiol..

[24] Marcobal A., de las Rivas B., Landete J.M., Tabera L., Munoz R. (2012). Tyramine and phenylethylamine biosynthesis by food bacteria. Crit. Rev. Food Sci. Nutr..

[25] Jairath G., Singh P.K., Dabur R.S., Rani M., Chaudhari M. (2015). Biogenic amines in meat and meat products and its public health significance: A review. J. Food Sci. Technol..

[26] Pircher A., Bauer F., Paulsen P. (2007). Formation of cadaverine, histamine, putrescine and tyramine by bacteria isolated from meat, fermented sausages and cheeses. Eur. Food Res. Technol..

[27] Suzzi G., Gardini F. (2003). Biogenic amines in dry fermented sausages: A review. Int. J. Food Microbiol..

[28] del Rio B., Redruello B., Linares D.M., Ladero V., Ruas-Madiedo P., Fernandez M., Martin M.C., Alvarez M.A. (2019). The biogenic amines putrescine and cadaverine show in vitro cytotoxicity at concentrations that can be found in foods. Sci. Rep..

[29] Bover-Cid S., Hugas M., Izquierdo-Pulido M., Vidal-Carou M.C. (2001). Amino acid-decarboxylase activity of bacteria isolated from fermented pork sausages. Int. J. Food Microbiol..

[30] Bover-Cid S., Torriani S., Gatto V., Tofalo R., Suzzi G., Belletti N., Gardini F. (2009). Relationships between microbial population dynamics and putrescine and cadaverine accumulation during dry fermented sausage ripening. J. Appl. Microbiol..

[31] Curiel J.A., Ruiz-Capillas C., de las Rivas B., Carrascosa A.V., Jiménez-Colmenero F., Muñoz R. (2011). Production of biogenic amines by lactic acid bacteria and enterobacteria isolated from fresh pork sausages packaged in different atmospheres and kept under refrigeration. Meat Sci..

[32] de las Rivas B., Ruiz-Capillas C., Carrascosa A.V., Curiel J.A., Jimenez-Colmenero F., Munoz R. (2008). Biogenic amine production by Gram-positive bacteria isolated from Spanish dry-cured “chorizo” sausage treated with high pressure and kept in chilled storage. Meat Sci..

[33] Komprda T., Sladkova P., Petirova E., Dohnal V., Burdychova R. (2010). Tyrosine- and histidine-decarboxylase positive lactic acid bacteria and enterococci in dry fermented sausages. Meat Sci..

[34] Durlu-Ozkaya F., Ayhan K., Vural N. (2001). Biogenic amines produced by *Enterobacteriaceae* isolated from meat products. Meat Sci..

[35] Lazaro C.A., Conte-Junior C.A., Canto A.C., Guerra Monteiro M.L., Costa-Lima B., da Cruz A.G., Marsico E.T., Franco R.M. (2015). Biogenic amines as bacterial quality indicators in different poultry meat species. LWT Food Sci. Technol..

[36] Li L., Wen X., Wen Z., Chen S., Wang L., Wei X. (2018). Evaluation of the biogenic amines formation and degradation abilities of *Lactobacillus curvatus* from Chinese bacon. Front. Microbiol..

[37] Masson F., Talon R., Montel M.C. (1996). Histamine and tyramine production by bacteria from meat products. Int. J. Food Microbiol..

[38] Landeta G., Curiel J.A., Carrascosa A.V., Munoz R., de las Rivas B. (2013). Characterization of coagulase-negative staphylococci isolated from Spanish dry cured meat products. Meat Sci..

[39] Landeta G., de las Rivas B., Carrascosa A.V., Muñoz R. (2007). Screening of biogenic amine production by coagulase-negative staphylococci isolated during industrial Spanish dry-cured ham processes. Meat Sci..

[40] Marty E., Bodenmann C., Buchs J., Hadorn R., Eugster-Meier E., Lacroix C., Meile L. (2012). Prevalence of antibiotic resistance in coagulase-negative staphylococci from spontaneously fermented meat products and safety assessment for new starters. Int. J. Food Microbiol..

[41] Seitter M., Geng B., Hertel C. (2011). Binding to extracellular matrix proteins and formation of biogenic amines by food-associated coagulase-negative staphylococci. Int. J. Food Microbiol..

[42] Simonova M., Strompfova V., Marcinakova M., Laukovda A., Vesterlund S., Moratalla M.L., Bover-Cid S., Vidal-Carou C. (2006). Characterization of *Staphylococcus xylosus* and *Staphylococcus carnosus* isolated from Slovak meat products. Meat Sci..

[43] Stavropoulou D.A., Borremans W., De Vuyst L., De Smet S., Leroy F. (2015). Amino acid conversions by coagulase-negative staphylococci in a rich medium: Assessment of inter- and intraspecies heterogeneity. Int. J. Food Microbiol..

[44] Coton E., Mulder N., Coton M., Pochet S., Trip H., Lolkema J.S. (2010). Origin of the putrescine-producing ability of the coagulase-negative bacterium *Staphylococcus epidermidis* 2015B. Appl. Environ. Microbiol..

[45] Braem G., De Vliegher S., Supré K., Haesebrouck F., Leroy F., De Vuyst L. (2011). (GTG)5-PCR fingerprinting for the classification and identification of coagulase-negative *Staphylococcus* species from bovine milk and teat apices: A comparison of type strains and field isolates. Vet. Microbiol..

[46] Tagg J.R., Bannister L.V. (1979). “Fingerprinting” β-haemolytic streptococci by their production of and sensitivity to bacteriocine-like inhibitors. J. Med. Microbiol..

[47] Lee N.-K., Jun S.-A., Ha J.-U., Paik H.-D. (2000). Screening and characterization of bacteriocinogenic lactic acid bacteria from Jeot-Gal, a Korean fermented fish food. J. Microbiol. Biotechnol..

[48] Clauwers C., Vanoirbeek K., Delbrassinne L., Michiels C.W. (2016). Construction of nontoxigenic mutants of nonproteolytic *Clostridium botulinum* NCTC 11219 by insertional mutagenesis and gene replacement. Appl. Environ. Microbiol..

[49] Gu Z., Eils R., Schlesner M. (2016). Complex heatmaps reveal patterns and correlations in multidimensional genomic data. Bioinformatics.

[50] Wickham H. (2016). ggplot2: Elegant Graphics for Data Analysis.

[51] R Core Team (2018). R: A Language and Environment for Statistical Computing.

[52] Stavropoulou D.A., De Maere H., Berardo A., Janssens B., Filippou P., De Vuyst L., De Smet S., Leroy F. (2018). Species pervasiveness within the group of coagulase-negative staphylococci associated with meat fermentation is modulated by pH. Front. Microbiol..

[53] Stavropoulou D.A., Van Reckem E., De Smet S., De Vuyst L., Leroy F. (2018). The narrowing down of inoculated communities of coagulase-negative staphylococci in fermented meat models is modulated by temperature and pH. Int. J. Food Microbiol..

[54] Coppola S., Mauriello G., Aponte M., Moschetti G., Villani F. (2000). Microbial succession during ripening of Naples-type salami, a southern Italian fermented sausage. Meat Sci..

[55] Stavropoulou D.A., De Maere H., Berardo A., Janssens B., Filippou P., De Vuyst L., De Smet S., Leroy F. (2018). Pervasiveness of *Staphylococcus carnosus* over *Staphylococcus xylosus* is affected by the level of acidification within a conventional meat starter culture set-up. Int. J. Food Microbiol..

[56] Otto M. (2009). *Staphylococcus epidermidis*—The “accidental” pathogen. Nat. Rev. Microbiol..

[57] Raz R., Colodner R., Kunin C.M. (2005). Who Are You—*Staphylococcus saprophyticus*?. Clin. Infect. Dis..

[58] Janek D., Zipperer A., Kulik A., Krismer B., Peschel A. (2016). High frequency and diversity of antimicrobial activities produced by nasal *Staphylococcus* strains against bacterial competitors. PLoS Pathog..

[59] Sandiford S., Upton M. (2012). Identification, characterization, and recombinant expression of epidermicin NI01, a novel unmodified bacteriocin produced by *Staphylococcus epidermidis* that displays potent activity against staphylococci. Antimicrob. Agents Chemother..

[60] Nascimento J.D.S., Coelho M.L.V., Ceotto H., Potter A., Fleming L.R., Salehian Z., Nes I.F., Bastos M.C.F. (2012). Genes involved in immunity to and secretion of aureocin A53, an atypical class II bacteriocin produced by *Staphylococcus aureus* A53. J. Bacteriol..

[61] Navaratna M.A.D.B., Sahl H.-G., Tagg J.R. (1999). Identification of genes encoding two-component lantibiotic production in *Staphylococcus aureus* C55 and other phage group II *S. aureus* strains and demonstration of an association with the exfoliative toxin B gene. Infect. Immun..

[62] Bennallack P.R., Burt S.R., Heder M.J., Robison R.A., Griffitts J.S. (2014). Characterization of a novel plasmid-borne thiopeptide gene cluster in *Staphylococcus epidermidis* strain 115. J. Bacteriol..

[63] Brito M.A.V.P., Somkuti G.A., Renye J.A. (2011). Production of antilisterial bacteriocins by staphylococci isolated from bovine milk. J. Dairy Sci..

[64] Rahmdel S., Shekarforoush S.S., Hosseinzadeh S., Torriani S., Gatto V. (2019). Antimicrobial spectrum activity of bacteriocinogenic *Staphylococcus* strains isolated from goat and sheep milk. J. Dairy Sci..

[65] Carson D.A., Barkema H.W., Naushad S., De Buck J. (2017). Bacteriocins of non-aureus staphylococci isolated from bovine milk. Appl. Environ. Microbiol..

[66] Brito M.A., Somkuti G.A., Renye J.A. (2011). Isolation of bacteriocin-producing staphylococci from Brazilian cheese. J. Food Saf..

[67] Hong J., Quan L.-H., Heu S., Jung K.S., Han S.-W., Moon E., Roh E. (2014). A new antimicrobial substance produced by *Staphylococcus pasteuri* isolated from vegetables. Food Sci. Biotechnol..

[68] Braem G., Stijlemans B., Van Haken W., De Vliegher S., De Vuyst L., Leroy F. (2014). Antibacterial activities of coagulase-negative staphylococci from bovine teat apex skin and their inhibitory effect on mastitis-related pathogens. J. Appl. Microbiol..

[69] Nascimento J.D.S., Fagundes P.C., Brito M.A.V.P., dos Santos K.R.N., Bastos M.C.F. (2005). Production of bacteriocins by coagulase-negative staphylococci involved in bovine mastitis. Vet. Microbiol..

[70] Nakatsuji T., Chen T.H., Narala S., Chun K.A., Two A.M., Yun T., Shafiq F., Kotol P.F., Bouslimani A., Melnik A.V. (2017). Antimicrobials from human skin commensal bacteria protect against *Staphylococcus aureus* and are deficient in atopic dermatitis. Sci. Transl. Med..

[71] Kleerebezem M. (2004). Quorum sensing control of lantibiotic production; nisin and subtilin autoregulate their own biosynthesis. Peptides.

[72] Kleerebezem M., Quadri L.E. (2001). Peptide pheromone-dependent regulation of antimicrobial peptide production in Gram-positive bacteria: A case of multicellular behavior. Peptides.

[73] Kleerebezem M., Kuipers O.P., de Vos W.M., Stiles M.E., Quadri L.E.N. (2001). A two-component signal-transduction cascade in *Carnobacterium piscicola* LV17B: Two signaling peptides and one sensor-transmitter. Peptides.

[74] Leroy F., de Vuyst L. (1999). Temperature and pH conditions that prevail during fermentation of sausages are optimal for production of the antilisterial bacteriocin sakacin K. Appl. Environ. Microbiol..

[75] de Sarrau B., Clavel T., Clerté C., Carlin F., Giniès C., Nguyen-The C. (2012). Influence of anaerobiosis and low temperature on *Bacillus cereus* growth, metabolism, and membrane properties. Appl. Environ. Microbiol..

[76] Kröckel L., Kongo J.M. (2013). The role of lactic acid bacteria in safety and flavour development of meat and meat products. Lactic Acid Bacteria—R & D for Food, Health and Livestock Purposes.

[77] Peypoux F., Bonmatin J.M., Wallach J. (1999). Recent trends in the biochemistry of surfactin. Appl. Microbiol. Biotechnol..

[78] Souto G.I., Correa O.S., Montecchia M.S., Kerber N.L., Pucheu N.L., Bachur M., García A.F. (2004). Genetic and functional characterization of a *Bacillus* sp. strain excreting surfactin and antifungal metabolites partially identified as iturin-like compounds. J. Appl. Microbiol..

[79] Tran T.T., Munita J.M., Arias C.A. (2015). Mechanisms of drug resistance: Daptomycin resistance. Ann. N. Y. Acad. Sci..

[80] Fair R.J., Tor Y. (2014). Antibiotics and bacterial resistance in the 21st century. Perspect. Med. Chem..

[81] Nemeghaire S., Argudín M.A., Feßler A.T., Hauschild T., Schwarz S., Butaye P. (2014). The ecological importance of the *Staphylococcus sciuri* species group as a reservoir for resistance and virulence genes. Vet. Microbiol..

[82] Zeman M., Mašlaňová I., Indráková A., Šiborová M., Mikulášek K., Bendíčková K., Plevka P., Vrbovská V., Zdráhal Z., Doškař J. (2017). *Staphylococcus sciuri* bacteriophages double-convert for staphylokinase and phospholipase, mediate interspecies plasmid transduction, and package mecA gene. Sci. Rep..

[83] Lucas P.M., Wolken W.A.M., Claisse O., Lolkema J.S., Lonvaud-Funel A. (2005). Histamine-producing pathway encoded on an unstable plasmid in *Lactobacillus hilgardii* 0006. Appl. Environ. Microbiol..

[84] Spano G., Russo P., Lonvaud-Funel A., Lucas P., Alexandre H., Grandvalet C., Coton E., Coton M., Barnavon L., Bach B. (2010). Biogenic amines in fermented foods. Eur. J. Clin. Nutr..

[85] Sánchez Mainar M., Weckx S., Leroy F. (2014). Coagulase-negative staphylococci favor conversion of arginine into ornithine despite a widespread genetic potential for nitric oxide synthase activity. Appl. Environ. Microbiol..

[86] Marcobal Á., de las Rivas B., García-Moruno E., Muñoz R. (2004). The tyrosine decarboxylation test does not differentiate *Enterococcus faecalis* from *Enterococcus faecium*. Syst. Appl. Microbiol..

[87] Aymerich T., Martín B., Garriga M., Vidal-Carou M.C., Bover-Cid S., Hugas M. (2006). Safety properties and molecular strain typing of lactic acid bacteria from slightly fermented sausages. J. Appl. Microbiol..

[88] Landete J.M., Pardo I., Ferrer S. (2007). Tyramine and phenylethylamine production among lactic acid bacteria isolated from wine. Int. J. Food Microbiol..

[89] Gardini F., Bover-Cid S., Tofalo R., Belletti N., Gatto V., Suzzi G., Torriani S. (2008). Modeling the aminogenic potential of *Enterococcus faecalis* EF37 in dry fermented sausages through chemical and molecular approaches. Appl. Environ. Microbiol..

[90] Ruiz-Capillas C., Herrero A.M. (2019). Impact of biogenic amines on food quality and safety. Foods.

[91] de las Rivas B., Rodríguez H., Carrascosa A.V., Muñoz R. (2008). Molecular cloning and functional characterization of a histidine decarboxylase from *Staphylococcus capitis*. J. Appl. Microbiol..

[92] Drosinos E.H., Paramithiotis S., Kolovos G., Tsikouras I., Metaxopoulos I. (2007). Phenotypic and technological diversity of lactic acid bacteria and staphylococci isolated from traditionally fermented sausages in Southern Greece. Food Microbiol..

[93] Seitter M., Nerz C., Rosenstein R., Goetz F., Hertel C. (2011). DNA microarray-based detection of genes involved in safety and technologically relevant properties of food associated coagulase-negative staphylococci. Int. J. Food Microbiol..

[94] Martin B., Garriga M., Hugas M., Bover-Cid S., Veciana-Nogues M.T., Aymerich T. (2006). Molecular, technological and safety characterization of Gram-positive catalase-positive cocci from slightly fermented sausages. Int. J. Food Microbiol..

[95] Parente E., Martuscelli M., Gardini F., Grieco S., Crudele M.A., Suzzi G. (2001). Evolution of microbial populations and biogenic amine production in dry sausages produced in Southern Italy. J. Appl. Microbiol..

[96] Bover-Cid S., Izquierdo-Pulido M., Vidal-Carou M.C. (2001). Effect of the interaction between a low tyramine-producing *Lactobacillus* and proteolytic staphylococci on biogenic amine production during ripening and storage of dry sausages. Int. J. Food Microbiol..

