# Supplementary material for: Exploring the Ambiguous Status of Coagulase-Negative Staphylococci in the Biosafety of Fermented Meats: The Case of Antibacterial Activity Versus Biogenic Amine Formation
Source: Microorganisms. 2020 Jan 24;8(2):167. doi: 10.3390/microorganisms8020167 (PMC7074764; doi:10.3390/microorganisms8020167)
Supplement: Supplementary file 1 [file microorganisms-08-00167-s001.pdf]

Supplementary materials

Exploring the ambiguous status of coagulase-negative staphylococci in the biosafety of fermented meats: the case of antibacterial activity *versus* biogenic amine formation

David Van der Veken [1], Rafik Benhachemi [2], Christina Charmpi [1], Lore Ockerman [1], Marijke Poortmans [2], Emiel Van Reckem [1], Chris Michiels [2] and Frédéric Leroy [1,*]

[1] Research Group of Industrial Microbiology and Food Biotechnology (IMDO), Faculty of Sciences and Bio-engineering Sciences, Vrije Universiteit Brussel, Brussels, Belgium; frederic.leroy@vub.be
[2] Laboratory of Food Microbiology and Leuven Food Science and Nutrition Research Centre (LFoRCe), KU Leuven, Leuven, Belgium; chris.michiels@biw.kuleuven.be
* Correspondence: frederic.leroy@vub.be

Table S1. Overview of the staphylococcal library used in this study.

ID	Species	Origin	Identification gene(s)	% of identity	Accession number
IMDO-S1	*S. epidermidis*	Teat apex skin			
IMDO-S2	*S. arlettae*	Teat apex skin			
IMDO-S3	*S. auricularis*	Teat apex skin			
IMDO-S4	*S. saprophyticus*	Fermented meat			
IMDO-S5	*S. carnosus*	Fermented meat			
IMDO-S6	*S. carnosus*	Fermented meat			
IMDO-S7	*S. carnosus*	Fermented meat			
IMDO-S8	*S. carnosus*	Starter culture			
IMDO-S9	*S. carnosus*	Starter culture			
IMDO-S10	*S. carnosus*	Starter culture	*rpob, tuf*	100	CP016760.1
IMDO-S11	*S. carnosus*	Starter culture			
IMDO-S12	*S. carnosus*	Starter culture			
IMDO-S13	*S. carnosus*	Starter culture			
IMDO-S14	*S. carnosus*	Fermented meat	*rpob, tuf*	100	CP016760.1
IMDO-S15	*S. carnosus*	Starter culture			
IMDO-S16	*S. carnosus*	Fermented meat			
IMDO-S17	*S. epidermidis*	Teat apex skin			
IMDO-S18	*S. arlettae*	Teat apex skin			
IMDO-S19	*S. cohnii*	Milk			
IMDO-S20	*S. cohnii*	Teat apex skin			
IMDO-S21	*S. cohnii*	Teat apex skin			
IMDO-S22	*Staphylococcus* sp.	Teat apex skin			
IMDO-S23	*Staphylococcus* sp.	Teat apex skin			

IMDO-S24	*Staphylococcus* sp.	Teat apex skin
IMDO-S25	*S. epidermidis*	Fermented meat
IMDO-S26	*S. epidermidis*	Unknown
IMDO-S27	*S. epidermidis*	Teat apex skin
IMDO-S28	*S. epidermidis*	Teat apex skin
IMDO-S29	*S. epidermidis*	Teat apex skin
IMDO-S30	*S. epidermidis*	Fermented meat
IMDO-S31	*S. equorum*	Milk
IMDO-S32	*S. equorum*	Teat apex skin
IMDO-S33	*S. equorum*	Fermented meat
IMDO-S34	*S. saprophyticus*	Fermented meat
IMDO-S35	*S. equorum*	Fermented meat
IMDO-S36	*S. equorum*	Fermented meat
IMDO-S37	*S. saprophyticus*	Fermented meat
IMDO-S38	*S. equorum*	Fermented meat
IMDO-S39	*S. equorum*	Fermented meat
IMDO-S40	*S. saprophyticus*	Fermented meat
IMDO-S41	*S. equorum*	Fermented meat
IMDO-S42	*S. equorum*	Fermented meat
IMDO-S43	*S. equorum*	Fermented meat
IMDO-S44	*S. equorum*	Fermented meat
IMDO-S45	*S. equorum*	Fermented meat
IMDO-S46	*S. carnosus*	Fermented meat
IMDO-S47	*S. fleuretti*	Milk
IMDO-S48	*S. carnosus*	Fermented meat
IMDO-S49	*S. haemolyticus*	Teat apex skin
IMDO-S50	*S. epidermidis*	Teat apex skin
IMDO-S51	*S. haemolyticus*	Fermented meat
IMDO-S52	*S. carnosus*	Fermented meat
IMDO-S53	*S. hominis*	Unknown
IMDO-S54	*S. saprophyticus*	Fermented meat
IMDO-S55	*S. pasteuri*	Fermented meat
IMDO-S56	*S. pasteuri*	Fermented meat
IMDO-S57	*S. pasteuri*	Fermented meat
IMDO-S58	*S. saprophyticus*	Milk
IMDO-S59	*S. saprophyticus*	Fermented meat
IMDO-S60	*S. carnosus*	Fermented meat
IMDO-S61	*S. saprophyticus*	Fermented meat
IMDO-S62	*S. saprophyticus*	Teat apex skin
IMDO-S63	*S. saprophyticus*	Teat apex skin
IMDO-S64	*S. saprophyticus*	Teat apex skin
IMDO-S65	*S. saprophyticus*	Fermented meat
IMDO-S66	*S. simulans*	Unknown
IMDO-S67	*S. succinus*	Fermented meat
IMDO-S68	*S. succinus*	Fermented meat

IMDO-S69	*S. succinus*	Fermented meat			
IMDO-S70	*S. sciuri*	Teat apex skin			
IMDO-S71	*S. cohnii*	Teat apex skin			
IMDO-S72	*S. sciuri*	Fermented meat			
IMDO-S73	*S. saprophyticus*	Milk			
IMDO-S74	*S. warneri*	Milk			
IMDO-S75	*S. equorum*	Teat apex skin	*rpob, tuf*	100	CP013980.1
IMDO-S76	*S. xylosus*	Starter culture			
IMDO-S77	*S. xylosus*	Milk			
IMDO-S78	*S. xylosus*	Milk			
IMDO-S79	*S. xylosus*	Fermented meat			
IMDO-S80	*S. xylosus*	Fermented meat			
IMDO-S81	*S. xylosus*	Fermented meat			
IMDO-S82	*S. xylosus*	Fermented meat			
IMDO-S83	*S. xylosus*	Teat apex skin			
IMDO-S84	*S. xylosus*	Teat apex skin			
IMDO-S85	*S. xylosus*	Fermented meat			
IMDO-S86	*S. xylosus*	Fermented meat			
IMDO-S87	*S. xylosus*	Fermented meat			
IMDO-S88	*S. xylosus*	Starter culture			
IMDO-S89	*S. succinus*	Unknown			
IMDO-S90	*S. warneri*	Fermented meat			
IMDO-S91	*S. saprophyticus*	Fermented meat			
IMDO-S92	*S. lugdunensis*	Fermented meat			
IMDO-S93	*S. epidermidis*	Fermented meat			
IMDO-S94	*S. epidermidis*	Fermented meat			
IMDO-S95	*S. simulans*	Fermented meat	*rpob, tuf*	100	CP015642.1
IMDO-S96	*S. pasteuri*	Fermented meat			
IMDO-S97	*S. aureus*	Fermented meat			
IMDO-S98	*S. epidermidis*	Fermented meat			
IMDO-S99	*S. epidermidis*	Fermented meat			
IMDO-S100	*S. aureus*	Fermented meat	*rpob, tuf*	100	CP035005.1
IMDO-S101	*S. epidermidis*	Fermented meat			
IMDO-S102	*S. xylosus*	Fermented meat			
IMDO-S103	*S. xylosus*	Fermented meat			
IMDO-S104	*S. xylosus*	Fermented meat			
IMDO-S105	*S. xylosus*	Fermented meat			
IMDO-S106	*S. xylosus*	Fermented meat			
IMDO-S107	*S. epidermidis*	Contamination			
IMDO-S108	*S. equorum*	Contamination			
IMDO-S109	*S. xylosus*	Contamination			
IMDO-S110	*S. equorum*	Contamination			
IMDO-S111	*S. xylosus*	Contamination			
IMDO-S112	*S. warneri*	Contamination			
IMDO-S113	*S. warneri*	Contamination			

IMDO-S114	*S. saprophyticus*	Raw pork meat			
IMDO-S115	*S. saprophyticus*	Raw pork meat			
IMDO-S116	*S. equorum*	Raw pork meat			
IMDO-S117	*S. saprophyticus*	Raw pork meat			
IMDO-S118	*S. equorum*	Raw pork meat			
IMDO-S119	*S. equorum*	Raw pork meat	*rpob, tuf*	100	CP013714.1
IMDO-S120	*S. aureus*	Raw pork meat	*rpob*	100	CP018100.1
IMDO-S121	*S. xylosus*	Raw pork meat			
IMDO-S122	*S. equorum*	Raw pork meat	*rpob, tuf*	99	CP013714.1
IMDO-S123	*S. xylosus*	Fermented meat	*rpoB*	99	CP008724.1
IMDO-S124	*S. aureus*	Fermented meat	*rpob, tuf*	100	CP035005.1
IMDO-S125	*S. xylosus*	Fermented meat	*rpob, tuf*	99	CP008724.1
IMDO-S126	*S. aureus*	Fermented meat	*rpob*	100	CP018100.1
IMDO-S127	*S. aureus*	Fermented meat	*rpob, tuf*	100	CP035005.1
IMDO-S128	*S. xylosus*	Fermented meat	*rpob*	100	CP008724.1
IMDO-S129	*S. xylosus*	Fermented meat			
IMDO-S130	*S. xylosus*	Fermented meat			
IMDO-S131	*S. equorum*	Fermented meat			
IMDO-S132	*S. xylosus*	Fermented meat			
IMDO-S133	*S. equorum*	Fermented meat	*rpoB*	100	CP013980.1
IMDO-S134	*S. xylosus*	Fermented meat			
IMDO-S135	*S. xylosus*	Fermented meat			
IMDO-S136	*S. equorum*	Fermented meat			
IMDO-S137	*S. xylosus*	Fermented meat			
IMDO-S138	*S. equorum*	Raw pork meat			
IMDO-S139	*S. epidermidis*	Raw pork meat			
IMDO-S140	*S. equorum*	Raw pork meat			
IMDO-S141	*S. equorum*	Raw pork meat			
IMDO-S142	*S. equorum*	Raw pork meat			
IMDO-S143	*S. equorum*	Raw pork meat			
IMDO-S144	*S. sciuri*	Raw pork meat			
IMDO-S145	*S. xylosus*	Raw pork meat			
IMDO-S146	*S. equorum*	Raw pork meat			
IMDO-S147	*S. equorum*	Fermented meat	*rpoB*	100	CP013980.1
IMDO-S148	*S. equorum*	Fermented meat			
IMDO-S149	*S. equorum*	Fermented meat			
IMDO-S150	*S. equorum*	Fermented meat	*rpoB*	100	CP013980.1
IMDO-S151	*S. equorum*	Fermented meat	*rpoB*	100	CP013980.1
IMDO-S152	*S. xylosus*	Fermented meat	*rpoB*	99	CP008724.1
IMDO-S153	*S. epidermidis*	Fermented meat	*rpob*	98	CP030246.1
IMDO-S154	*S. xylosus*	Fermented meat	*rpob*	100	LN554884.1
IMDO-S155	*S. xylosus*	Fermented meat			
IMDO-S156	*S. equorum*	Fermented meat			
IMDO-S157	*S. saprophyticus*	Fermented meat	*rpob*	100	CP022093.2
IMDO-S158	*S. xylosus*	Fermented meat			

IMDO-S159	*S. xylosus*	Fermented meat	*rpoB*	99	CP008724.1
IMDO-S160	*S. equorum*	Fermented meat	*rpoB*	100	CP013980.1
IMDO-S161	*S. xylosus*	Fermented meat			
IMDO-S162	*S. xylosus*	Fermented meat			
IMDO-S163	*S. equorum*	Fermented meat			
IMDO-S164	*S. equorum*	Fermented meat			
IMDO-S165	*S. xylosus*	Fermented meat			
IMDO-S166	*S. equorum*	Fermented meat	*rpoB*	100	CP013980.1
IMDO-S167	*S. xylosus*	Fermented meat			
IMDO-S168	*S. epidermidis*	Fermented meat			
IMDO-S169	*S. equorum*	Fermented meat	*rpoB*	100	CP013980.1
IMDO-S170	*S. xylosus*	Fermented meat			
IMDO-S171	*S. equorum*	Fermented meat			
IMDO-S172	*S. equorum*	Fermented meat	*rpoB*	100	CP013980.1
IMDO-S173	*S. equorum*	Fermented meat	*rpoB*	100	CP013980.1
IMDO-S174	*S. xylosus*	Fermented meat			
IMDO-S175	*S. equorum*	Fermented meat	*rpoB*	100	CP013980.1
IMDO-S176	*S. equorum*	Fermented meat	*rpob, tuf*	100	CP008724.1
IMDO-S177	*S. xylosus*	Fermented meat	*rpoB*	99	CP008724.1
IMDO-S178	*S. equorum*	Fermented meat			
IMDO-S179	*S. xylosus*	Fermented meat	*rpoB*	99	CP008724.1
IMDO-S180	*S. xylosus*	Fermented meat			
IMDO-S181	*S. equorum*	Fermented meat			
IMDO-S182	*S. equorum*	Fermented meat			
IMDO-S183	*S. epidermidis*	Fermented meat	*rpoB*	99	CP009046.1
IMDO-S184	*S. equorum*	Raw pork meat			
IMDO-S185	*S. saprophyticus*	Raw pork meat			
IMDO-S186	*S. capitis*	Raw pork meat	*rpoB*	100	CP007601.1
IMDO-S187	*S. equorum*	Raw pork meat			
IMDO-S188	*S. saprophyticus*	Raw pork meat			
IMDO-S189	*S. equorum*	Raw pork meat			
IMDO-S190	*S. equorum*	Raw pork meat	*rpob*	100	CP018100.1
IMDO-S191	*S. equorum*	Raw pork meat	*tuf*	100	CP013714.1
IMDO-S192	*S. equorum*	Raw pork meat			
IMDO-S193	*S. xylosus*	Fermented meat	*rpoB*	100	CP008724.1
IMDO-S194	*S. xylosus*	Fermented meat	*rpoB*	99	CP008724.1
IMDO-S195	*S. xylosus*	Fermented meat			
IMDO-S196	*S. equorum*	Fermented meat			
IMDO-S197	*S. equorum*	Fermented meat			
IMDO-S198	*S. xylosus*	Fermented meat			
IMDO-S199	*S. equorum*	Fermented meat			
IMDO-S200	*S. xylosus*	Fermented meat	*rpoB*	99	CP008724.1
IMDO-S201	*S. xylosus*	Fermented meat			
IMDO-S202	*S. equorum*	Fermented meat			
IMDO-S203	*S. xylosus*	Fermented meat			

IMDO-S204	*S. xylosus*	Fermented meat			
IMDO-S205	*S. equorum*	Fermented meat			
IMDO-S206	*S. xylosus*	Fermented meat	*rpoB*	100	CP008724.1
IMDO-S207	*S. xylosus*	Fermented meat			
IMDO-S208	*S. equorum*	Fermented meat			
IMDO-S209	*S. xylosus*	Fermented meat			
IMDO-S210	*S. equorum*	Fermented meat			
IMDO-S211	*S. equorum*	Fermented meat	*rpob*	99	CP018100.1
IMDO-S212	*S. xylosus*	Fermented meat			
IMDO-S213	*S. xylosus*	Fermented meat			
IMDO-S214	*S. xylosus*	Fermented meat			
IMDO-S215	*S. xylosus*	Fermented meat	*rpoB*	100	CP008724.1
IMDO-S216	*S. xylosus*	Fermented meat			
IMDO-S217	*S. equorum*	Fermented meat			
IMDO-S218	*S. xylosus*	Fermented meat			
IMDO-S219	*S. equorum*	Fermented meat	*rpob*	100	CP018100.1
IMDO-S220	*S. saprophyticus*	Fermented meat			
IMDO-S221	*S. equorum*	Fermented meat			
IMDO-S222	*S. pasteuri*	Fermented meat	*tuf*	99	CP017463.1
IMDO-S223	*S. equorum*	Fermented meat			
IMDO-S224	*S. xylosus*	Fermented meat			
IMDO-S225	*S. equorum*	Fermented meat			
IMDO-S226	*S. equorum*	Fermented meat			
IMDO-S227	*S. equorum*	Fermented meat	*rpob*	100	CP018100.1
IMDO-S228	*S. xylosus*	Raw pork meat			
IMDO-S229	*S. simulans*	Raw pork meat	*tuf*	100	CP015642.1
IMDO-S230	*S. saprophyticus*	Raw pork meat	*rpob*	100	CP014113.2
IMDO-S231	*S. equorum*	Raw pork meat			
IMDO-S232	*S. equorum*	Raw pork meat	*rpob*	100	CP018100.1
IMDO-S233	*S. equorum*	Raw pork meat	*rpob*	100	CP018100.1
IMDO-S234	*S. saprophyticus*	Raw pork meat	*rpob*	100	CP014113.2
IMDO-S235	*S. equorum*	Raw pork meat			
IMDO-S236	*S. equorum*	Raw pork meat			
IMDO-S237	*S. epidermidis*	Fermented meat	*rpob*	100	CP009046.1
IMDO-S238	*S. xylosus*	Fermented meat			
IMDO-S239	*S. equorum*	Fermented meat			
IMDO-S240	*S. epidermidis*	Fermented meat	*rpob, tuf*	100	CP034115.1
IMDO-S241	*S. saprophyticus*	Fermented meat	*rpob*	100	CP014113.2
IMDO-S242	*S. xylosus*	Fermented meat			
IMDO-S243	*S. saprophyticus*	Fermented meat	*rpob*	100	CP014113.2
IMDO-S244	*S. xylosus*	Fermented meat	*rpob*	100	CP008724.1
IMDO-S245	*S. equorum*	Fermented meat			
IMDO-S246	*S. xylosus*	Fermented meat			
IMDO-S247	*S. saprophyticus*	Fermented meat	*rpob*	100	CP014113.2
IMDO-S248	*S. epidermidis*	Fermented meat			

IMDO-S249	*S. xylosus*	Fermented meat			
IMDO-S250	*S. equorum*	Fermented meat			
IMDO-S251	*S. xylosus*	Fermented meat			
IMDO-S252	*S. equorum*	Fermented meat	*rpob*	100	CP018100.1
IMDO-S253	*S. xylosus*	Fermented meat			
IMDO-S254	*S. epidermidis*	Fermented meat	*rpob*	100	CP009046.1
IMDO-S255	*S. xylosus*	Fermented meat			
IMDO-S256	*S. equorum*	Fermented meat	*rpob*	99	CP018100.1
IMDO-S257	*S. equorum*	Fermented meat	*rpob*	99	CP018100.1
IMDO-S258	*S. xylosus*	Fermented meat			
IMDO-S259	*S. xylosus*	Fermented meat			
IMDO-S260	*S. equorum*	Fermented meat	*rpob*	99	CP018100.1
IMDO-S261	*S. vitulinus*	Fermented meat			
IMDO-S262	*S. xylosus*	Fermented meat			
IMDO-S263	*S. equorum*	Fermented meat	*rpob, tuf*	100	CP013980.1
IMDO-S264	*S. xylosus*	Fermented meat			
IMDO-S265	*S. equorum*	Fermented meat	*rpob*	99	CP018100.1
IMDO-S266	*S. xylosus*	Fermented meat			
IMDO-S267	*S. equorum*	Fermented meat			
IMDO-S268	*S. equorum*	Fermented meat			
IMDO-S269	*S. xylosus*	Fermented meat			
IMDO-S270	*S. equorum*	Fermented meat			
IMDO-S271	*S. equorum*	Fermented meat			
IMDO-S272	*S. xylosus*	Fermented meat			
IMDO-S273	*S. equorum*	Fermented meat	*rpob*	100	CP018100.1
IMDO-S274	*S. equorum*	Fermented meat			
IMDO-S275	*S. xylosus*	Fermented meat			
IMDO-S276	*S. saprophyticus*	Raw pork meat			
IMDO-S277	*S. equorum*	Raw pork meat			
IMDO-S278	*S. equorum*	Raw pork meat	*rpob*	100	CP018100.1
IMDO-S279	*S. xylosus*	Raw pork meat			
IMDO-S280	*S. equorum*	Raw pork meat	*rpob*	100	CP018100.1
IMDO-S281	*S. equorum*	Raw pork meat			
IMDO-S282	*S. equorum*	Raw pork meat			
IMDO-S283	*S. xylosus*	Raw pork meat			
IMDO-S284	*S. equorum*	Raw pork meat			
IMDO-S285	*S. saprophyticus*	Fermented meat			
IMDO-S286	*S. saprophyticus*	Fermented meat	*rpob*	100	CP014113.2
IMDO-S287	*S. xylosus*	Fermented meat			
IMDO-S288	*S. epidermidis*	Fermented meat	*rpob*	100	CP009046.1
IMDO-S289	*S. xylosus*	Fermented meat	*rpob*	100	CP008724.1
IMDO-S290	*S. xylosus*	Fermented meat			
IMDO-S291	*S. epidermidis*	Fermented meat			
IMDO-S292	*S. xylosus*	Fermented meat	*rpob*	100	CP008724.1
IMDO-S293	*S. epidermidis*	Fermented meat	*rpob*	99	CP009046.1

IMDO-S294	*S. xylosus*	Fermented meat	*rpob*	100	CP008724.1
IMDO-S295	*S. equorum*	Fermented meat			
IMDO-S296	*S. equorum*	Fermented meat			
IMDO-S297	*S. xylosus*	Fermented meat	*rpob, tuf*	100	LN554884.1
IMDO-S298	*S. equorum*	Fermented meat	*rpob*	99	CP018100.1
IMDO-S299	*S. epidermidis*	Fermented meat			
IMDO-S300	*S. equorum*	Fermented meat	*rpob*	100	CP018100.1
IMDO-S301	*S. xylosus*	Fermented meat	*rpob*	100	LN554884.1
IMDO-S302	*S. epidermidis*	Fermented meat			
IMDO-S303	*S. epidermidis*	Fermented meat			
IMDO-S304	*S. saprophyticus*	Fermented meat			
IMDO-S305	*S. epidermidis*	Fermented meat	*rpob*	100	CP009046.1
IMDO-S306	*S. equorum*	Fermented meat			
IMDO-S307	*S. xylosus*	Fermented meat			
IMDO-S308	*S. epidermidis*	Fermented meat			
IMDO-S309	*S. epidermidis*	Fermented meat	*rpob*	100	CP009046.1
IMDO-S310	*S. xylosus*	Fermented meat	*rpob*	99	CP008724.1
IMDO-S311	*S. saprophyticus*	Fermented meat	*rpob*	100	CP014113.2
IMDO-S312	*S. xylosus*	Fermented meat			
IMDO-S313	*S. equorum*	Fermented meat			
IMDO-S314	*S. equorum*	Fermented meat			
IMDO-S315	*S. equorum*	Fermented meat			
IMDO-S316	*S. equorum*	Fermented meat			
IMDO-S317	*S. equorum*	Fermented meat	*rpob*	100	CP018100.1
IMDO-S318	*S. equorum*	Fermented meat			
IMDO-S319	*S. xylosus*	Fermented meat			
IMDO-S320	*S. xylosus*	Fermented meat			
IMDO-S321	*S. equorum*	Fermented meat			
IMDO-S322	*S. equorum*	Fermented meat			
IMDO-S323	*S. equorum*	Fermented meat			
IMDO-S324	*S. epidermidis*	Fermented meat	*rpob*	99	CP009046.1
IMDO-S325	*S. equorum*	Fermented meat			
IMDO-S326	*S. xylosus*	Fermented meat			
IMDO-S327	*S. equorum*	Fermented meat	*rpob*	100	CP018100.1
IMDO-S328	*S. saprophyticus*	Fermented meat			
IMDO-S329	*S. saprophyticus*	Contamination	*rpob, tuf*	100	CP035005.1
IMDO-S335	*S. xylosus*	Contamination	*tuf*	100	CP008724.1
IMDO-S336	*S. epidermidis*	Contamination	*rpob*	100	CP030246.1
IMDO-S337	*S. epidermidis*	Contamination	*rpob*	100	CP030246.1

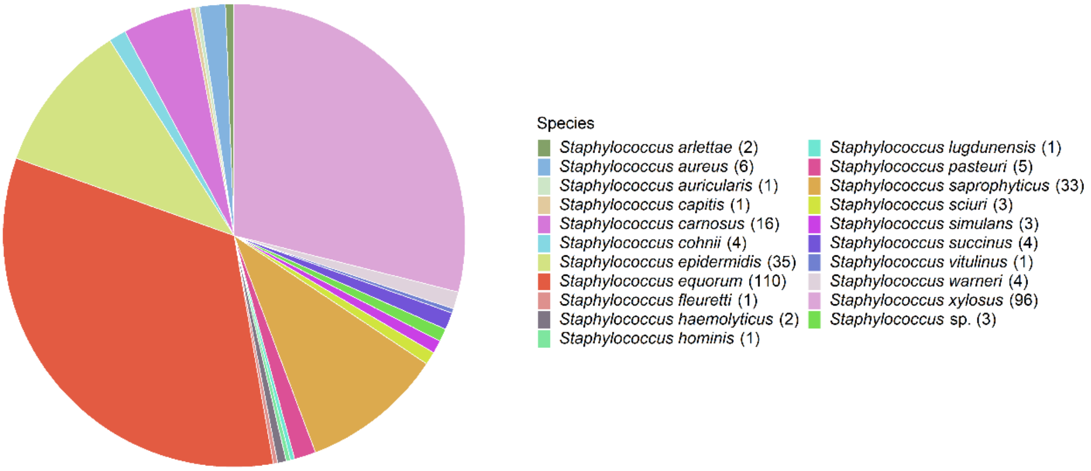

Figure S1. Species diversity of the staphylococcal strain collection (332 strains). The amount of strains per species is indicated by the number between brackets. Three isolates could not be identified to species level and were categorized as *Staphylococcus* sp.

Table S2. Scores of antibacterial activities of the staphylococcal library towards seven staphylococcal indicator strains. 0 = no inhibition, 1 = IZ ≤ 1 mm, 2 = 1 mm ≤ IZ ≤ 4 mm, 3 = 4 mm ≤ IZ ≤ 8 mm, 4 = IZ ≥ 8 mm. Antibacterial strains are highlighted in gray. Strains that were used in the negative set are indicated with an asterix (*).

**Name**	**Species**	**IMDO-S30** *S. epidermidis*	**IMDO-S16** *S. carnosus*	**IMDO-S62** *S. saprophyticus*	**IMDO-S73** *S. saprophyticus*	**IMDO-S86** *S. xylosus*	**IMDO-S32** *S. equorum*	**IMDO-S18** *S. arlettae*
IMDO-S1*	*S. epidermidis*	0	0	0	0	0	0	0
IMDO-S2	*S. arlettae*	0	0	0	0	0	0	0
IMDO-S3	*S. auricularis*	0	0	0	0	0	0	0
IMDO-S4	*S. saprophyticus*	0	0	0	0	0	0	0
IMDO-S5*	*S. carnosus*	0	0	0	0	0	0	0
IMDO-S6	*S. carnosus*	0	0	0	0	0	0	0
IMDO-S7	*S. carnosus*	0	0	0	0	0	0	0
IMDO-S8	*S. carnosus*	0	0	0	0	0	0	0
IMDO-S9*	*S. carnosus*	0	0	0	0	0	0	0
IMDO-S10	*S. carnosus*	0	0	0	0	0	0	0
IMDO-S11	*S. carnosus*	0	0	0	0	0	0	0
IMDO-S12	*S. carnosus*	0	0	0	0	0	0	0
IMDO-S13	*S. carnosus*	0	0	0	0	0	0	0
IMDO-S14	*S. carnosus*	0	0	0	0	0	0	0
IMDO-S15	*S. carnosus*	0	0	0	0	0	0	0
IMDO-S16	*S. carnosus*	0	0	0	0	0	0	0
IMDO-S17	*S. epidermidis*	0	0	0	0	0	0	0
IMDO-S18	*S. arlettae*	0	0	0	0	0	0	0
IMDO-S19	*S. cohnii*	0	0	0	0	0	0	0
IMDO-S20	*S. cohnii*	0	0	0	0	0	0	0
IMDO-S21	*S. cohnii*	0	0	0	0	0	0	0
IMDO-S22	*Staphylococcus* sp.	0	0	0	0	0	0	0
IMDO-S23	*Staphylococcus* sp.	0	0	0	0	0	0	0

IMDO-S24	*Staphylococcus* sp.	0	0	0	0	0	0	0
IMDO-S25	*S. epidermidis*	0	0	0	0	0	0	0
IMDO-S26	*S. epidermidis*	0	0	2	0	1	3	0
IMDO-S27	*S. epidermidis*	0	0	0	0	0	0	0
IMDO-S28	*S. epidermidis*	0	0	0	0	0	0	0
IMDO-S29	*S. epidermidis*	2	2	3	2	2	4	2
IMDO-S30	*S. epidermidis*	0	0	0	0	0	0	0
IMDO-S31*	*S. equorum*	0	0	0	0	0	0	0
IMDO-S32*	*S. equorum*	0	0	0	0	0	0	0
IMDO-S33	*S. equorum*	0	0	0	0	0	0	0
IMDO-S34*	*S. saprophyticus*	0	0	0	0	0	0	0
IMDO-S35	*S. equorum*	0	0	0	0	0	0	0
IMDO-S36	*S. equorum*	0	0	0	0	0	0	0
IMDO-S37	*S. saprophyticus*	0	0	0	0	0	0	0
IMDO-S38	*S. equorum*	0	0	0	0	0	0	0
IMDO-S39	*S. equorum*	0	0	0	0	0	0	0
IMDO-S40	*S. saprophyticus*	0	0	0	0	0	0	0
IMDO-S41	*S. equorum*	0	0	0	0	0	0	0
IMDO-S42	*S. equorum*	0	0	0	0	0	0	0
IMDO-S43	*S. equorum*	0	0	0	0	0	0	0
IMDO-S44	*S. equorum*	0	0	0	0	0	0	0
IMDO-S45	*S. equorum*	0	0	0	0	0	0	0
IMDO-S46	*S. carnosus*	0	0	0	0	0	0	0
IMDO-S47	*S. fleuretti*	1	0	2	1	2	2	2
IMDO-S48	*S. carnosus*	0	0	0	0	0	0	0
IMDO-S49	*S. haemolyticus*	0	0	0	0	0	0	0
IMDO-S50	*S. epidermidis*	0	0	0	0	0	0	0
IMDO-S51	*S. haemolyticus*	0	0	0	0	0	0	0
IMDO-S52	*S. carnosus*	0	0	0	0	0	0	0
IMDO-S53	*S. hominis*	0	0	0	0	0	0	0

IMDO-S54	*S. saprophyticus*	0	0	0	0	0	0	0
IMDO-S55	*S. pasteuri*	0	0	0	0	0	0	0
IMDO-S56	*S. pasteuri*	0	0	0	0	0	0	0
IMDO-S57	*S. pasteuri*	0	0	0	0	0	0	0
IMDO-S58*	*S. saprophyticus*	0	0	0	0	0	0	0
IMDO-S59	*S. saprophyticus*	0	0	0	0	0	0	0
IMDO-S60	*S. carnosus*	0	0	0	0	0	0	0
IMDO-S61	*S. saprophyticus*	0	0	0	0	0	0	0
IMDO-S62	*S. saprophyticus*	0	0	0	0	0	0	0
IMDO-S63*	*S. saprophyticus*	0	0	0	0	0	0	0
IMDO-S64	*S. saprophyticus*	0	0	0	0	0	0	0
IMDO-S65	*S. saprophyticus*	0	0	0	0	0	0	0
IMDO-S66	*S. simulans*	0	0	0	0	0	0	0
IMDO-S67	*S. succinus*	0	0	0	0	0	0	0
IMDO-S68	*S. succinus*	0	0	0	0	0	0	0
IMDO-S69	*S. succinus*	0	0	0	0	0	0	0
IMDO-S70*	*S. sciuri*	0	0	0	0	0	0	0
IMDO-S71	*S. cohnii*	0	0	0	0	0	0	0
IMDO-S72	*S. sciuri*	3	4	3	3	3	4	2
IMDO-S73	*S. saprophyticus*	0	0	0	0	0	0	0
IMDO-S74	*S. warneri*	0	0	0	0	0	0	0
IMDO-S75	*S. equorum*	0	0	0	0	0	0	0
IMDO-S76*	*S. xylosus*	0	0	0	0	0	0	0
IMDO-S77	*S. xylosus*	0	0	0	0	0	0	0
IMDO-S78*	*S. xylosus*	0	0	0	0	0	0	0
IMDO-S79	*S. xylosus*	0	0	0	0	0	0	0
IMDO-S80	*S. xylosus*	0	0	0	0	0	0	0
IMDO-S81	*S. xylosus*	0	0	0	0	0	0	0
IMDO-S82	*S. xylosus*	0	0	0	0	0	0	0
IMDO-S83*	*S. xylosus*	0	0	0	0	0	0	0

IMDO-S84	*S. xylosus*	0	0	0	0	0	0	0
IMDO-S85	*S. xylosus*	0	0	0	0	0	0	0
IMDO-S86	*S. xylosus*	0	0	0	0	0	0	2
IMDO-S87	*S. xylosus*	0	0	0	0	0	0	0
IMDO-S88	*S. xylosus*	0	0	0	0	0	0	0
IMDO-S89	*S. succinus*	0	0	0	0	0	0	0
IMDO-S90	*S. warneri*	0	0	0	0	0	0	0
IMDO-S91	*S. saprophyticus*	0	0	0	0	0	0	0
IMDO-S92	*S. lugdunensis*	0	0	0	0	0	0	0
IMDO-S93	*S. epidermidis*	0	0	0	0	0	0	0
IMDO-S94	*S. epidermidis*	0	0	0	0	0	0	0
IMDO-S95	*S. simulans*	0	0	0	0	0	0	0
IMDO-S96	*S. pasteuri*	0	0	0	0	0	0	0
IMDO-S97	*S. aureus*	0	0	0	0	0	0	1
IMDO-S98*	*S. epidermidis*	0	0	0	0	0	0	0
IMDO-S99	*S epidermidis*	0	0	0	0	0	0	0
IMDO-S100	*S. aureus*	0	0	0	0	0	0	0
IMDO-S101	*S. epidermidis*	0	0	2	0	0	0	0
IMDO-S102	*S. xylosus*	0	0	0	0	0	0	0
IMDO-S103	*S. xylosus*	0	0	0	0	0	0	0
IMDO-S104	*S. xylosus*	0	0	0	0	0	0	0
IMDO-S105	*S. xylosus*	0	0	0	0	0	0	0
IMDO-S106	*S. xylosus*	0	0	0	0	0	0	0
IMDO-S107	*S. epidermidis*	0	0	0	0	0	0	0
IMDO-S108	*S. equorum*	0	0	0	0	0	0	0
IMDO-S109	*S. xylosus*	0	0	0	0	0	0	0
IMDO-S110	*S. equorum*	0	0	1	2	1	0	0
IMDO-S111	*S. xylosus*	0	0	0	0	0	0	0
IMDO-S112	*S. warneri*	0	0	0	0	0	0	0
IMDO-S113	*S. warneri*	0	0	0	0	0	0	0

IMDO-S114	*S. saprophyticus*	0	0	0	0	0	0	0
IMDO-S115	*S. saprophyticus*	0	0	0	0	0	0	0
IMDO-S116	*S. equorum*	0	0	0	0	0	0	0
IMDO-S117	*S. saprophyticus*	0	0	0	0	0	0	0
IMDO-S118	*S. equorum*	0	0	0	0	0	0	0
IMDO-S119	*S. equorum*	0	0	0	0	0	0	0
IMDO-S120	*S. aureus*	0	0	0	0	0	0	0
IMDO-S121	*S. xylosus*	0	0	0	0	0	0	0
IMDO-S122	*S. equorum*	0	0	0	0	0	0	0
IMDO-S123	*S. xylosus*	0	0	0	1	0	4	0
IMDO-S124	*S. aureus*	0	0	0	0	0	0	0
IMDO-S125	*S. xylosus*	0	0	0	0	0	0	0
IMDO-S126	*S. aureus*	0	0	0	0	0	0	0
IMDO-S127	*S. aureus*	0	0	0	0	0	0	0
IMDO-S128	*S. xylosus*	0	0	0	0	0	0	0
IMDO-S129	*S. xylosus*	0	0	0	0	0	0	0
IMDO-S130	*S. xylosus*	0	0	0	0	0	0	0
IMDO-S131	*S. equorum*	0	0	0	1	0	4	0
IMDO-S132	*S. xylosus*	0	0	0	0	0	0	0
IMDO-S133	*S. equorum*	0	0	0	0	0	0	0
IMDO-S134	*S. xylosus*	0	0	0	0	0	0	0
IMDO-S135	*S. xylosus*	0	0	0	0	0	0	0
IMDO-S136	*S. equorum*	0	0	0	0	0	0	0
IMDO-S137	*S. xylosus*	0	0	0	0	0	0	0
IMDO-S138	*S. equorum*	0	0	0	0	0	0	0
IMDO-S139	*S. epidermidis*	0	0	0	0	0	0	0
IMDO-S140	*S. equorum*	0	0	0	0	0	0	0
IMDO-S141	*S. equorum*	0	0	0	0	0	0	0
IMDO-S142	*S. equorum*	0	0	0	0	0	0	0
IMDO-S143	*S. equorum*	0	0	0	0	0	0	0

IMDO-S144*	*S. sciuri*	0	0	0	0	0	0	0
IMDO-S145	*S. xylosus*	0	0	0	0	0	0	0
IMDO-S146	*S. equorum*	0	0	0	0	0	0	0
IMDO-S147	*S. equorum*	0	0	0	0	0	0	0
IMDO-S148	*S. equorum*	0	0	0	0	0	0	0
IMDO-S149	*S. equorum*	0	0	0	0	0	0	0
IMDO-S150	*S. equorum*	0	0	0	0	0	0	0
IMDO-S151	*S. equorum*	0	0	0	0	0	0	0
IMDO-S152	*S. xylosus*	0	0	0	1	1	0	0
IMDO-S153	*S. epidermidis*	0	0	0	0	0	0	0
IMDO-S154	*S. xylosus*	0	0	0	0	0	0	0
IMDO-S155	*S. xylosus*	0	0	0	1	1	0	0
IMDO-S156	*S. equorum*	0	0	0	0	0	0	0
IMDO-S157	*S. saprophyticus*	0	0	0	0	0	0	0
IMDO-S158	*S. xylosus*	0	0	0	0	0	0	0
IMDO-S159	*S. xylosus*	0	0	0	0	0	0	0
IMDO-S160	*S. equorum*	0	0	0	0	0	0	0
IMDO-S161	*S. xylosus*	0	0	0	0	0	0	0
IMDO-S162	*S. xylosus*	0	0	0	0	0	0	0
IMDO-S163	*S. equorum*	0	0	0	0	0	0	0
IMDO-S164	*S. equorum*	0	0	0	0	0	0	0
IMDO-S165	*S. xylosus*	0	0	0	0	0	0	0
IMDO-S166	*S. equorum*	0	0	0	0	0	0	0
IMDO-S167	*S. xylosus*	0	0	0	0	0	0	0
IMDO-S168	*S. epidermidis*	0	0	0	0	0	0	0
IMDO-S169	*S. equorum*	0	0	0	0	0	0	0
IMDO-S170	*S. xylosus*	0	0	0	0	0	0	0
IMDO-S171	*S. equorum*	0	0	0	0	0	0	0
IMDO-S172	*S. equorum*	0	0	0	0	0	0	0
IMDO-S173	*S. equorum*	0	0	0	0	0	0	0

IMDO-S174	*S. xylosus*	0	0	0	0	0	0	0
IMDO-S175	*S. equorum*	0	0	0	0	0	0	0
IMDO-S176	*S. equorum*	0	0	0	0	0	0	0
IMDO-S177	*S. xylosus*	0	0	0	0	0	0	0
IMDO-S178	*S. equorum*	0	0	0	0	0	0	0
IMDO-S179	*S. xylosus*	0	0	0	0	0	0	0
IMDO-S180	*S. xylosus*	0	0	0	0	0	0	0
IMDO-S181	*S. equorum*	0	0	0	0	0	0	0
IMDO-S182	*S. equorum*	0	0	0	0	0	0	0
IMDO-S183	*S. epidermidis*	0	0	0	0	0	0	0
IMDO-S184	*S. equorum*	0	0	0	0	0	0	0
IMDO-S185	*S. saprophyticus*	0	0	0	0	0	0	0
IMDO-S186	*S. capitis*	0	0	0	0	0	0	0
IMDO-S187	*S. equorum*	0	0	0	0	0	0	0
IMDO-S188	*S. saprophyticus*	0	0	0	0	0	0	0
IMDO-S189	*S. equorum*	0	0	0	0	0	0	0
IMDO-S190	*S. equorum*	0	0	0	0	0	0	0
IMDO-S191	*S. equorum*	0	0	0	0	0	0	0
IMDO-S192	*S. equorum*	0	0	0	0	0	0	0
IMDO-S193	*S. xylosus*	0	0	0	0	0	0	0
IMDO-S194	*S. xylosus*	0	0	0	0	0	0	0
IMDO-S195	*S. xylosus*	0	0	0	0	0	0	0
IMDO-S196	*S. equorum*	0	0	0	0	0	0	0
IMDO-S197	*S. equorum*	0	0	0	0	0	0	0
IMDO-S198	*S. xylosus*	0	0	0	0	0	0	0
IMDO-S199	*S. equorum*	0	0	0	0	0	0	0
IMDO-S200	*S. xylosus*	0	0	0	1	0	0	0
IMDO-S201	*S. xylosus*	0	0	0	0	0	0	0
IMDO-S202	*S. equorum*	0	0	0	0	0	0	0
IMDO-S203*	*S. xylosus*	0	0	0	0	0	0	0

IMDO-S204	*S. xylosus*	0	0	0	0	0	0	0
IMDO-S205	*S. equorum*	0	0	0	1	0	0	0
IMDO-S206	*S. xylosus*	0	0	0	0	0	0	0
IMDO-S207	*S. xylosus*	0	0	0	0	0	0	0
IMDO-S208	*S. equorum*	0	0	0	0	0	0	0
IMDO-S209	*S. xylosus*	0	0	0	0	0	0	0
IMDO-S210	*S. equorum*	0	0	0	0	0	0	0
IMDO-S211	*S. equorum*	0	0	0	0	0	0	0
IMDO-S212	*S. xylosus*	0	0	0	0	0	0	0
IMDO-S213	*S. xylosus*	0	0	0	0	0	0	0
IMDO-S214	*S. xylosus*	0	0	0	0	0	0	0
IMDO-S215	*S. xylosus*	0	0	0	0	0	0	0
IMDO-S216	*S. xylosus*	4	2	3	2	3	0	0
IMDO-S217	*S. equorum*	0	0	0	0	0	0	0
IMDO-S218	*S. xylosus*	0	0	0	0	0	0	0
IMDO-S219	*S. equorum*	0	0	0	0	0	0	0
IMDO-S220	*S. saprophyticus*	0	0	0	0	0	0	0
IMDO-S221	*S. equorum*	0	0	0	0	0	0	0
IMDO-S222	*S. pasteuri*	0	0	0	0	0	0	0
IMDO-S223	*S. equorum*	0	0	0	0	0	0	0
IMDO-S224	*S. xylosus*	0	0	0	0	0	4	0
IMDO-S225	*S. equorum*	0	0	0	0	0	0	0
IMDO-S226	*S. equorum*	0	0	0	0	0	0	0
IMDO-S227	*S. equorum*	0	0	0	0	0	0	0
IMDO-S228	*S. xylosus*	0	0	0	0	0	0	0
IMDO-S229	*S. simulans*	0	0	0	0	0	0	0
IMDO-S230	*S. saprophyticus*	0	0	0	0	0	0	0
IMDO-S231	*S. equorum*	0	0	0	0	0	0	0
IMDO-S232	*S. equorum*	0	0	0	0	0	0	0
IMDO-S233	*S. equorum*	0	0	0	0	0	0	0

IMDO-S234	*S. saprophyticus*	0	0	0	0	0	0	0
IMDO-S235	*S. equorum*	0	0	0	0	0	0	0
IMDO-S236	*S. equorum*	0	0	0	0	0	0	0
IMDO-S237	*S. epidermidis*	0	0	0	0	0	0	0
IMDO-S238	*S. xylosus*	0	0	0	0	0	0	0
IMDO-S239	*S. equorum*	0	0	0	0	0	0	0
IMDO-S240	*S. epidermidis*	0	0	0	0	0	0	0
IMDO-S241	*S. saprophyticus*	0	0	0	0	0	0	0
IMDO-S242	*S. xylosus*	0	0	0	0	0	0	0
IMDO-S243	*S. saprophyticus*	0	0	0	0	0	0	0
IMDO-S244	*S. xylosus*	0	0	0	0	0	0	0
IMDO-S245	*S. equorum*	0	0	0	0	0	0	0
IMDO-S246	*S. xylosus*	0	0	0	0	0	0	0
IMDO-S247	*S. saprophyticus*	0	0	0	0	0	0	0
IMDO-S248	*S. epidermidis*	0	0	0	0	0	0	0
IMDO-S249	*S. xylosus*	0	0	0	0	0	0	0
IMDO-S250	*S. equorum*	0	0	0	0	0	0	0
IMDO-S251	*S. xylosus*	0	0	0	0	0	0	0
IMDO-S252*	*S. equorum*	0	0	0	0	0	0	0
IMDO-S253	*S. xylosus*	0	0	0	0	0	0	0
IMDO-S254	*S. epidermidis*	0	0	0	0	0	0	0
IMDO-S255	*S. xylosus*	0	0	0	0	0	0	0
IMDO-S256	*S. equorum*	0	0	0	0	0	0	0
IMDO-S257	*S. equorum*	0	0	2	0	0	0	0
IMDO-S258	*S. xylosus*	0	0	0	0	0	0	0
IMDO-S259	*S. xylosus*	0	0	0	0	0	0	0
IMDO-S260	*S. equorum*	0	0	0	0	0	0	0
IMDO-S261	*S. vitulinus*	0	0	0	0	0	0	0
IMDO-S262	*S. xylosus*	0	0	0	0	0	0	0
IMDO-S263	*S. equorum*	0	0	0	0	0	0	0

IMDO-S264	*S. xylosus*	0	0	0	0	0	0	0
IMDO-S265	*S. equorum*	0	0	0	0	0	0	0
IMDO-S266	*S. xylosus*	0	0	0	0	0	0	0
IMDO-S267	*S. equorum*	0	0	0	0	0	0	0
IMDO-S268	*S. equorum*	0	0	0	0	0	0	0
IMDO-S269	*S. xylosus*	0	0	0	0	0	0	0
IMDO-S270	*S. equorum*	0	0	0	0	0	0	0
IMDO-S271	*S. equorum*	0	0	0	0	0	0	0
IMDO-S272	*S. xylosus*	0	0	0	0	0	0	0
IMDO-S273	*S. equorum*	0	0	0	0	0	0	0
IMDO-S274	*S. equorum*	0	0	0	0	0	0	0
IMDO-S275	*S. xylosus*	0	0	0	0	0	0	0
IMDO-S276	*S. saprophyticus*	0	0	0	0	0	0	0
IMDO-S277	*S. equorum*	0	0	0	0	0	0	0
IMDO-S278	*S. equorum*	0	0	0	0	0	0	0
IMDO-S279	*S. xylosus*	0	0	0	0	0	0	0
IMDO-S280	*S. equorum*	0	0	0	0	0	0	0
IMDO-S281	*S. equorum*	0	0	0	0	0	0	0
IMDO-S282	*S. equorum*	0	0	0	0	0	0	0
IMDO-S283	*S. xylosus*	0	0	0	0	0	0	0
IMDO-S284	*S. equorum*	0	0	0	0	0	0	0
IMDO-S285	*S. saprophyticus*	0	0	0	0	0	0	0
IMDO-S286	*S. saprophyticus*	0	0	0	1	1	2	1
IMDO-S287	*S. xylosus*	0	0	0	0	0	0	0
IMDO-S288	*S. epidermidis*	0	0	0	0	0	0	0
IMDO-S289	*S. xylosus*	0	0	0	0	0	0	0
IMDO-S290	*S. xylosus*	0	0	0	0	0	0	0
IMDO-S291	*S. epidermidis*	0	0	0	0	0	0	0
IMDO-S292	*S. xylosus*	0	0	0	0	0	0	0
IMDO-S293	*S. epidermidis*	0	0	0	0	0	0	0

IMDO-S294	*S. xylosus*	0	0	0	0	0	0	0
IMDO-S295	*S. equorum*	0	0	0	0	0	0	0
IMDO-S296	*S. equorum*	0	0	0	0	0	0	0
IMDO-S297	*S. xylosus*	0	0	0	0	0	0	0
IMDO-S298	*S. equorum*	0	0	0	0	0	0	0
IMDO-S299	*S. epidermidis*	0	0	0	0	0	0	0
IMDO-S300	*S. equorum*	0	0	0	0	0	0	0
IMDO-S301	*S. xylosus*	0	0	0	0	0	0	0
IMDO-S302	*S. epidermidis*	0	0	0	0	0	0	0
IMDO-S303	*S. epidermidis*	0	0	0	0	0	0	0
IMDO-S304	*S. saprophyticus*	0	0	0	0	0	0	0
IMDO-S305	*S. epidermidis*	0	0	0	0	0	0	0
IMDO-S306	*S. equorum*	0	0	0	0	0	0	0
IMDO-S307	*S. xylosus*	0	0	0	0	0	0	0
IMDO-S308	*S. epidermidis*	0	0	0	0	0	0	0
IMDO-S309	*S. epidermidis*	0	0	0	0	0	0	0
IMDO-S310	*S. xylosus*	0	0	0	0	0	0	0
IMDO-S311	*S. saprophyticus*	0	0	0	0	0	0	0
IMDO-S312	*S. xylosus*	0	0	0	0	0	0	0
IMDO-S313	*S. equorum*	0	0	0	0	0	0	0
IMDO-S314	*S. equorum*	0	0	0	0	0	0	0
IMDO-S315	*S. equorum*	0	0	0	0	0	0	0
IMDO-S316	*S. equorum*	0	0	0	0	0	0	0
IMDO-S317	*S. equorum*	0	0	0	0	0	0	0
IMDO-S318	*S. equorum*	0	0	0	0	0	0	0
IMDO-S319	*S. xylosus*	0	0	0	0	0	0	0
IMDO-S320	*S. xylosus*	0	0	0	0	0	0	0
IMDO-S321	*S. equorum*	0	0	0	0	0	0	0
IMDO-S322	*S. equorum*	0	0	0	0	0	0	0
IMDO-S323	*S. equorum*	0	0	0	0	0	0	0

IMDO-S324	*S. epidermidis*	0	0	0	0	0	0	0
IMDO-S325	*S. equorum*	0	0	0	0	0	0	0
IMDO-S326	*S. xylosus*	0	0	0	0	0	0	0
IMDO-S327	*S. equorum*	0	0	0	0	0	0	0
IMDO-S328	*S. saprophyticus*	0	0	0	0	0	0	0
IMDO-S329	*S. saprophyticus*	0	0	0	0	0	0	0
IMDO-S335	*S. xylosus*	0	0	0	0	0	0	0
IMDO-S336	*S. epidermidis*	0	0	0	0	0	0	0
IMDO-S337	*S. epidermidis*	0	0	0	0	0	0	0

Table S3. List of non-toxigenic *Clostridium botulinum* group I and group II used as indicator strains in spot-on-lawn assay.

***Clostridium botulinum* group**	**Strain**	**Origin**
I	WHH2	White herring
	SDBE2.1	Sludge/mud
	MEL	Milk powder
	ME2.1	Manure
	TR2.2	Mud
	BB32	Mud
	ZBS 1	Sand
	ZO1.2	Beach sand
	SDE2.2	Sludge/mud
II	Δ8266	Non-toxigenic construct[1]
	Δ11219	Non-toxigenic construct[1]
	ME2.2	Dried manure
	ZBS 2	Pike, intestine
	ZBS 3	Bream
	ZBS 4	Lake sediment

II	ZBS 5	Horse, faeces
	ZBS 6	Human, faeces
	ZBS 7	Cattle, rumen content
	ZBS 8	Cattle, faeces
	ZBS 9	Liver sausage
	ZBS 11	Lake sediment
	ZBS 12	Cattle, rumen content
	ZBS 13	Cattle, faeces
	ZBS 14	Mallart duck, duodenum
	ZBS 15	Biogas plant
	ZBS 17	Silage
	ZBS 18	Human, stomach content
	ZBS 19	Honey
	ZBS 20	Honey
	3676	Food isolate
	3677	Food isolate
	3678	Food isolate
	DSM 1985	Lake sediment

[1][48]

Table S4. Spot-on-lawn assay testing the antibacterial impact of *Staphylococcus sciuri* IMDO-S72 on different indicator strains. + Or -: inhibition or no inhibition of the indicator strain.

**Gram-staining category**	**Indicator strains**	**Antibacterial activity *S. sciuri* IMDO-S72**
+	*Listeria monocytogenes* SCOTT A	+
	Bacillus cereus 14579	-
	B. cereus AFSSA_08CEB44bac	+
	B. cereus INRA KBAAD5	+
	B. cereus INRA PA	+

	B. cereus INRA SB′	+
	B. cereus TZ415	+
**+**	*Bacillus subtilis* 168	+
	Lactococcus lactis IL1402	+
	Lactobacillus sakei	+
	Staphylococcus aureus IMDO-S100	+
**-**	*Escherichia coli* DH5α	-
	Salmonella enterica Typhimurium LT2	-

Table S5. Biogenic amine concentrations of the staphylococcal library after 7 and 14 d of incubation in BHI measured using UPLC-MS/MS. Strains are ordered alphabetically, the names of the biogenic amines are abbreviated: AGM = agmatine, CAD = cadaverine, HIS = histamine, PEA = β-phenylethylamine, PUT = putrescine, SPD = spermidine, SPM = spermine, TRY = tryptamine and TYR = tyramine. Concentrations were assumed zero if they were under the limit of quantification (ULQ).

		**7 d**									**14 d**								
Species	Strain ID	AGM	CAD	HIS	PEA	PUT	SPD	SPM	TRY	TYR	AGM	CAD	HIS	PEA	PUT	SPD	SPM	TRY	TYR
S. arlettae	2	8.60	36.42	1.31	0.00	50.67	38.60	30.46	0.00	0.00	1.60	20.71	0.00	0.00	32.58	37.99	28.32	0.00	0.00
S. arlettae	18	6.82	23.52	0.00	0.00	30.05	33.00	29.32	0.00	0.00	3.22	8.32	0.00	0.00	19.43	33.60	25.61	0.00	0.00
S. aureus	97	10.19	26.14	0.00	0.00	41.42	35.93	28.59	0.00	0.00	8.50	23.09	0.00	0.00	46.31	46.96	29.32	0.00	0.00
S. aureus	100	11.53	38.55	1.15	0.00	58.16	40.23	29.84	0.00	0.00	7.29	19.25	0.00	0.00	35.65	42.40	27.89	0.00	0.00
S. aureus	120	0.00	0.00	0.00	2.79	0.00	9.25	19.50	0.00	469.64	12.16	31.89	0.00	614.78	41.72	41.49	26.03	5.85	10221.80
S. aureus	124	16.68	39.42	12.20	198.09	123.43	12.27	42.65	0.00	18181.61	14.21	22.39	0.00	676.48	33.94	21.49	53.46	5.44	7451.22
S. aureus	126	13.44	29.20	3.10	170.06	42.28	11.09	29.93	0.00	7973.31	9.85	11.95	0.00	631.67	20.25	19.31	39.04	4.43	6447.40
S. aureus	127	197.35	1913.42	22.27	404.02	18119.89	13.68	25.08	0.00	0.00	1.84	1804.41	28.18	643.43	28701.70	16.30	42.09	0.00	0.00
S. auricularis	3	6.73	34.73	0.00	0.00	43.92	41.86	29.56	0.00	0.00	6.20	21.21	0.00	0.00	25.25	41.28	26.96	0.00	0.00
S. capitis	186	8.86	56.51	8029.75	1026.43	44.84	30.86	23.38	19.52	6759.93	7.73	76.15	8763.09	1557.45	45.47	33.98	22.07	35.90	6657.03
S. carnosus	5	10.35	32.80	3.41	805.56	45.43	42.47	27.38	179.10	221.22	9.75	27.27	0.00	1426.90	40.22	44.17	24.74	333.05	377.11

S. carnosus	6	6.36	26.73	0.00	720.00	37.36	40.66	26.39	143.73	175.43	5.54	14.12	0.00	1694.86	22.16	41.79	23.09	350.82	354.17
S. carnosus	7	10.01	31.36	3.40	784.22	41.70	43.66	27.60	170.93	222.18	9.38	23.02	4.37	1705.99	29.32	49.47	71.86	363.84	381.02
S. carnosus	8	10.09	36.75	2.48	759.72	47.55	43.99	27.53	165.66	223.06	8.96	21.58	0.00	1559.62	28.19	44.07	46.00	315.51	341.95
S. carnosus	9	9.00	31.35	1.53	706.63	38.86	42.81	27.74	152.90	193.16	5.28	17.63	0.00	1367.98	24.22	43.75	33.57	280.07	281.15
S. carnosus	10	8.32	27.02	0.38	695.08	31.95	42.45	28.66	143.47	189.67	7.09	17.80	0.00	1378.58	25.19	42.40	29.86	270.28	299.28
S. carnosus	11	13.06	39.91	4.27	781.24	61.33	44.51	28.43	180.33	273.85	9.71	23.57	0.00	1551.28	29.79	44.11	28.95	337.74	355.23
S. carnosus	12	9.46	38.14	5.86	807.28	56.24	45.28	28.29	180.92	256.13	4.57	11.88	0.00	1636.37	20.63	41.41	26.84	316.88	291.75
S. carnosus	13	8.95	37.55	5.74	796.81	54.97	44.36	27.86	188.49	290.01	6.95	18.18	0.00	1430.50	30.34	41.04	24.91	305.42	390.31
S. carnosus	14	8.94	33.04	3.80	674.21	47.57	44.29	29.48	190.04	148.91	4.96	14.86	0.00	1011.52	23.01	41.51	25.59	249.77	158.05
S. carnosus	15	7.05	31.76	3.77	735.72	48.71	41.12	27.11	170.17	237.48	7.24	20.94	0.00	1697.35	31.14	43.95	26.02	374.35	436.55
S. carnosus	16	6.10	23.68	0.00	667.81	38.75	39.29	25.72	131.96	155.53	5.57	17.14	0.00	1514.25	23.79	42.60	24.76	274.10	316.99
S. carnosus	46	6.69	28.33	2.40	712.37	44.32	39.88	28.12	148.45	204.53	6.01	13.08	0.00	1888.42	17.35	40.96	22.99	424.99	441.47
S. carnosus	48	9.27	30.46	1.68	732.93	42.61	38.22	24.90	165.70	209.04	8.58	18.81	0.00	1676.91	30.80	41.23	23.19	382.92	397.26
S. carnosus	52	9.91	36.55	3.71	654.27	56.05	40.34	26.35	147.59	242.86	7.57	20.18	0.00	1764.85	31.61	41.48	27.18	393.23	441.74
S. carnosus	60	10.85	36.50	6.18	645.64	50.65	38.16	25.02	155.49	246.40	8.50	15.92	0.00	1479.37	33.65	35.66	23.19	323.03	376.90
S. cohnii	19	9.37	28.20	2.93	0.00	41.72	31.54	28.46	0.00	0.00	6.70	19.69	0.00	0.00	33.95	29.09	25.03	0.00	0.00
S. cohnii	20	7.91	31.32	1.08	0.00	44.12	35.74	26.38	0.00	0.00	4.32	15.45	0.00	0.00	24.20	34.68	25.09	0.00	0.00
S. cohnii	21	8.26	24.26	0.55	0.00	38.11	35.89	29.19	0.00	0.00	7.30	18.88	0.00	0.00	34.45	36.95	27.35	0.00	0.00
S. cohnii	71	7.14	28.99	2.42	0.00	39.89	26.79	26.96	0.00	0.00	8.89	23.52	0.00	0.00	32.98	24.26	25.49	0.00	0.00
S. epidermidis	1	10.45	40.81	2.11	0.00	57.54	44.89	30.96	0.00	0.00	8.90	29.19	0.00	0.00	43.82	46.51	28.40	0.00	0.00
S. epidermidis	17	9.75	33.95	1.99	0.00	48.32	39.19	31.06	0.00	0.00	8.12	24.54	0.00	0.00	43.96	40.86	28.61	0.00	0.00
S. epidermidis	25	7.01	23.28	0.00	0.00	31.33	32.78	28.82	0.00	0.00	8.07	21.75	0.00	0.00	36.98	40.65	30.42	0.00	0.00
S. epidermidis	26	10.12	29.83	15.10	884.73	39.52	25.78	25.77	174.71	113.75	8.78	23.13	15.01	1445.78	41.54	27.00	27.04	302.45	186.37
S. epidermidis	27	7.91	32.14	2.81	0.00	38.26	34.72	29.72	0.00	0.00	6.72	19.42	0.00	0.00	32.43	39.57	28.37	0.00	0.00
S. epidermidis	28	9.67	30.35	0.00	0.00	43.00	35.43	28.21	0.00	0.00	7.55	18.18	0.00	0.00	31.68	38.48	30.68	0.00	0.00
S. epidermidis	29	10.44	35.47	2.91	0.00	55.60	36.79	30.64	0.00	0.00	7.04	17.00	0.00	0.00	32.83	39.80	29.02	0.00	0.00
S. epidermidis	30	8.24	28.37	0.00	0.00	40.42	36.82	29.55	0.00	0.00	6.52	16.70	0.00	0.00	31.00	43.44	28.41	0.00	0.00
S. epidermidis	50	7.96	22.44	0.00	0.00	34.39	29.52	28.26	0.00	0.00	10.56	24.14	0.00	0.00	37.60	37.10	65.93	0.00	0.00
S. epidermidis	93	12.87	193.17	3.26	0.00	55.84	36.67	29.60	0.00	0.00	8.66	432.85	0.00	0.00	37.40	37.14	27.06	0.00	0.00
S. epidermidis	94	7.79	36.42	0.00	0.00	35.83	30.48	27.21	0.00	0.00	7.44	49.68	0.00	0.00	36.83	34.17	26.58	0.00	0.00

S. epidermidis	98	8.89	32.77	0.00	0.00	47.14	38.50	30.21	0.00	0.00	9.48	27.60	0.00	0.00	43.38	46.03	29.29	0.00	0.00
S. epidermidis	99	11.12	37.72	3.06	0.00	49.92	31.20	28.58	0.00	0.00	8.87	22.02	0.00	0.00	33.46	30.87	26.91	0.00	0.00
S. epidermidis	101	10.22	27.72	11.09	851.18	38.48	24.89	25.71	168.35	105.45	11.36	23.89	11.27	1373.02	45.17	24.45	23.98	313.40	226.12
S. epidermidis	107	6.29	24.12	0.00	0.00	37.76	30.94	26.45	0.00	0.00	4.79	14.88	0.00	0.00	26.70	33.86	25.26	0.00	0.00
S. epidermidis	139	7.73	25.84	0.00	0.00	37.71	33.96	28.58	0.00	0.00	7.40	16.67	0.00	0.00	27.80	34.08	26.83	0.00	0.00
S. epidermidis	153	10.24	23.97	0.00	171.28	30.71	10.31	28.22	0.00	7116.74	12.13	25.30	0.00	818.87	41.28	18.03	25.24	7.29	7941.20
S. epidermidis	168	6.05	19.77	0.00	0.00	32.52	25.74	25.98	0.00	0.00	5.38	16.37	0.00	0.00	28.20	29.73	24.63	0.00	0.00
S. epidermidis	183	7.38	24.86	0.00	0.00	33.53	34.21	27.77	0.00	0.00	6.70	25.15	0.00	0.00	37.08	38.92	26.59	0.00	0.00
S. epidermidis	237	9.66	23.06	0.00	0.00	32.58	30.64	34.07	0.00	0.00	10.49	31.80	0.00	0.00	56.10	38.43	28.80	0.00	0.00
S. epidermidis	240	8.20	20.75	0.00	0.00	27.00	26.74	27.13	0.00	0.00	5.53	14.52	0.00	0.00	23.53	28.00	22.51	0.00	0.00
S. epidermidis	248	13.31	31.11	0.00	0.00	50.22	33.17	31.83	0.00	0.00	9.03	24.55	0.00	0.00	45.98	34.61	26.94	0.00	0.00
S. epidermidis	254	7.83	14.06	0.00	0.00	22.01	29.76	27.13	0.00	0.00	5.58	17.57	0.00	0.00	27.96	34.61	25.80	0.00	0.00
S. epidermidis	288	10.00	23.75	0.23	0.00	37.91	35.73	28.03	0.00	0.00	10.02	25.88	0.00	0.00	41.55	37.81	26.73	0.00	0.00
S. epidermidis	291	12.50	29.56	0.06	0.00	41.89	35.37	27.82	0.00	0.00	9.32	19.57	0.00	0.00	31.85	39.63	26.50	0.00	0.00
S. epidermidis	293	13.72	27.39	0.00	0.00	34.92	33.55	29.09	0.00	0.00	11.26	24.79	0.00	0.00	40.42	38.27	28.39	0.00	0.00
S. epidermidis	299	8.11	25.21	0.00	0.00	30.32	33.46	27.78	0.00	0.00	7.82	22.01	0.00	0.00	32.93	39.12	27.94	0.00	0.00
S. epidermidis	302	8.65	20.74	0.00	0.00	27.25	31.96	27.23	0.00	0.00	8.21	26.71	0.00	0.00	34.58	40.11	26.87	0.00	0.00
S. epidermidis	303	7.75	19.57	0.00	0.00	28.20	30.09	26.91	0.00	0.00	9.47	24.36	0.00	0.00	36.62	37.40	27.71	0.00	0.00
S. epidermidis	305	11.14	21.98	0.00	0.00	35.17	32.99	28.08	0.00	0.00	7.38	23.79	0.00	0.00	36.07	41.01	28.89	0.00	0.00
S. epidermidis	308	7.70	16.79	0.00	0.00	18.94	30.92	26.52	0.00	0.00	13.99	42.74	0.00	0.00	75.74	44.42	33.35	0.00	0.00
S. epidermidis	309	10.84	26.23	0.00	0.00	35.64	33.01	27.80	0.00	0.00	7.78	19.51	0.00	0.00	33.34	36.33	28.85	0.00	0.00
S. epidermidis	324	8.23	22.00	0.00	0.00	29.07	31.22	27.19	0.00	0.00	6.79	14.82	0.00	0.00	23.88	36.25	31.18	0.00	0.00
S. epidermidis	336	10.41	27.25	0.00	0.00	39.39	32.16	26.68	0.00	0.00	6.87	15.15	0.00	0.00	25.36	39.53	29.00	0.00	0.00
S. epidermidis	337	10.45	24.86	0.00	0.00	35.94	32.20	28.29	0.00	0.00	6.14	13.06	0.00	0.00	24.09	35.70	28.69	0.00	0.00
S. equorum	31	7.61	23.04	0.00	0.00	28.22	32.50	29.15	0.00	0.00	7.26	14.25	0.00	0.00	26.48	33.81	25.28	0.00	0.00
S. equorum	32	7.01	25.06	1.15	0.00	35.70	39.12	29.04	0.00	0.00	6.61	20.73	0.00	0.00	37.79	40.55	26.82	0.00	0.00
S. equorum	33	8.67	26.59	0.21	0.00	31.07	32.95	29.35	0.00	0.00	7.54	20.90	0.00	0.00	33.86	31.31	25.66	0.00	0.00
S. equorum	35	9.08	26.82	0.00	0.00	35.70	31.39	27.11	0.00	0.00	7.47	17.73	0.00	0.00	30.53	36.29	28.72	0.00	0.00
S. equorum	36	9.06	30.68	1.13	0.00	47.44	29.09	27.48	0.00	0.00	4.26	13.62	0.00	0.00	23.20	30.88	26.08	0.00	0.00
S. equorum	38	8.55	33.65	1.95	0.00	50.41	21.23	27.85	0.00	0.00	4.75	9.42	0.00	0.00	16.41	21.11	23.70	0.00	0.00

S. equorum	39	7.88	24.77	0.00	0.00	37.05	32.64	29.76	0.00	0.00	8.12	23.54	0.00	0.00	38.19	30.62	25.72	0.00	0.00
S. equorum	41	10.33	39.98	5.48	0.00	65.24	34.09	31.15	0.00	0.00	5.28	17.31	0.00	0.00	27.80	30.10	25.54	0.00	0.00
S. equorum	42	7.33	24.36	0.00	0.00	35.16	30.93	28.95	0.00	0.00	6.45	19.08	0.00	0.00	33.17	34.13	25.52	0.00	0.00
S. equorum	43	8.08	30.21	3.89	0.00	40.83	31.32	29.54	0.00	0.00	6.47	19.15	0.00	0.00	30.15	30.52	26.79	0.00	0.00
S. equorum	44	5.17	27.58	0.42	0.00	39.48	37.40	28.24	0.00	0.00	7.99	22.88	0.00	0.00	34.34	42.20	27.03	0.00	0.00
S. equorum	45	8.57	28.21	2.28	0.00	40.82	16.11	28.54	0.00	0.00	7.55	19.30	0.00	0.00	29.10	19.20	24.80	0.00	0.00
S. equorum	75	7.84	29.26	1.11	0.00	40.96	33.39	27.42	0.00	0.00	7.80	20.02	0.00	0.00	34.10	35.32	25.50	0.00	0.00
S. equorum	108	10.55	29.65	3.69	0.00	43.45	28.43	26.20	0.00	0.00	6.43	10.72	0.00	0.00	23.72	29.40	23.14	0.00	0.00
S. equorum	110	9.83	37.63	4.44	0.00	45.20	26.89	28.32	0.00	0.00	4.39	13.41	0.00	0.00	23.08	26.79	25.74	0.00	0.00
S. equorum	116	9.14	36.61	1.86	0.00	50.95	28.18	27.39	0.00	0.00	5.33	15.76	0.00	0.00	28.25	28.71	24.95	0.00	0.00
S. equorum	118	9.54	15.91	0.00	0.00	22.07	32.43	35.36	0.00	0.00	7.72	20.48	0.00	0.00	31.40	33.72	24.19	0.00	0.00
S. equorum	119	7.86	26.69	0.00	0.00	40.14	36.00	35.72	0.00	0.00	8.93	18.18	0.00	0.00	36.35	40.29	28.24	0.00	0.00
S. equorum	122	8.81	26.54	0.00	0.00	39.04	25.82	31.37	0.00	0.00	6.84	18.00	0.00	0.00	30.04	28.25	24.63	0.00	0.00
S. equorum	131	77.15	32.22	2.10	0.00	47.40	21.72	27.41	0.00	0.00	145.85	16.27	0.00	0.00	28.43	31.60	27.01	0.00	0.00
S. equorum	133	7.39	28.48	1.28	0.00	43.97	26.82	25.14	0.00	0.00	6.93	19.03	0.00	0.00	31.46	26.74	25.02	0.00	0.00
S. equorum	136	11.06	39.97	3.74	0.00	57.73	30.89	29.39	0.00	0.00	7.45	18.48	0.00	0.00	38.60	28.41	25.48	0.00	0.00
S. equorum	138	8.02	31.98	3.20	0.00	44.75	27.85	29.10	0.00	0.00	5.56	18.04	0.00	0.00	28.62	27.84	25.49	0.00	0.00
S. equorum	140	5.88	23.65	0.00	0.00	31.80	24.46	25.66	0.00	0.00	5.45	16.46	0.00	0.00	22.27	28.76	26.22	0.00	0.00
S. equorum	141	8.18	27.63	0.00	0.00	37.61	28.42	27.69	0.00	0.00	7.05	19.98	0.00	0.00	30.08	31.95	26.85	0.00	0.00
S. equorum	142	10.87	37.89	2.11	0.00	60.60	26.26	27.63	0.00	0.00	6.95	21.16	0.00	0.00	32.85	29.21	26.91	0.00	0.00
S. equorum	143	9.50	31.49	3.08	0.00	43.02	27.29	29.75	0.00	0.00	6.24	23.82	0.00	0.00	36.28	29.24	28.61	0.00	0.00
S. equorum	146	5.32	17.79	0.00	0.00	25.00	26.44	27.81	0.00	0.00	6.92	18.22	0.00	0.00	31.99	30.35	27.59	0.00	0.00
S. equorum	147	9.10	25.91	1.66	0.00	34.47	35.12	28.34	0.00	0.00	4.87	13.82	0.00	0.00	22.46	35.19	25.42	0.00	0.00
S. equorum	148	9.13	28.39	0.00	0.00	42.36	27.53	27.72	0.00	0.00	7.41	19.84	0.00	0.00	31.64	31.50	25.76	0.00	0.00
S. equorum	149	12.15	39.64	4.09	0.00	53.81	31.61	30.85	0.00	0.00	6.28	18.36	0.00	0.00	30.18	32.29	24.80	0.00	0.00
S. equorum	150	6.95	23.74	0.00	0.00	37.27	27.32	27.41	0.00	0.00	4.96	14.34	0.00	0.00	20.88	27.98	25.28	0.00	0.00
S. equorum	151	11.48	39.78	2.99	0.00	64.14	29.62	30.35	0.00	0.00	6.98	20.70	0.00	0.00	28.84	27.69	26.21	0.00	0.00
S. equorum	156	12.11	37.11	5.59	0.00	59.96	29.83	28.84	0.00	0.00	5.92	18.90	0.00	0.00	31.99	28.65	24.79	0.00	0.00
S. equorum	160	8.67	24.72	0.00	0.00	34.06	33.63	28.29	0.00	0.00	5.61	15.39	0.00	0.00	30.33	34.25	24.72	0.00	0.00
S. equorum	163	13.38	45.26	5.14	0.00	68.62	34.13	31.00	0.00	0.00	7.73	21.58	0.00	0.00	40.73	34.30	25.82	0.00	0.00

S. equorum	164	9.81	28.68	0.54	0.00	44.44	31.88	29.75	0.00	0.00	6.83	21.42	0.00	0.00	32.43	43.25	29.90	0.00	0.00
S. equorum	166	8.44	32.70	1.54	0.00	50.86	26.42	27.38	0.00	0.00	9.01	28.53	0.00	0.00	50.00	29.41	26.41	0.00	0.00
S. equorum	169	7.26	21.47	0.00	0.00	30.55	29.84	27.33	0.00	0.00	6.80	24.41	0.00	0.00	38.02	32.04	26.73	0.00	0.00
S. equorum	171	8.42	25.52	1.77	0.00	33.62	29.68	25.77	0.00	0.00	6.55	15.35	0.00	0.00	24.82	28.08	22.42	0.00	0.00
S. equorum	172	8.40	22.04	0.00	0.00	31.66	27.78	26.24	0.00	0.00	5.17	16.32	0.00	0.00	31.73	29.29	24.51	0.00	0.00
S. equorum	173	12.34	38.55	2.64	0.00	56.76	29.18	29.32	0.00	0.00	8.12	26.35	0.00	0.00	38.30	28.33	25.32	0.00	0.00
S. equorum	175	6.81	20.64	0.00	0.00	30.65	31.05	26.50	0.00	0.00	7.66	29.14	0.00	0.00	40.54	38.49	26.25	0.00	0.00
S. equorum	176	11.95	39.18	6.09	0.00	56.44	26.65	31.40	0.00	0.00	8.78	28.41	0.00	0.00	42.47	28.29	26.59	0.00	0.00
S. equorum	178	7.21	20.91	0.00	0.00	23.68	26.67	28.88	0.00	0.00	4.84	19.25	0.00	0.00	31.60	27.17	26.00	0.00	0.00
S. equorum	181	8.15	31.75	0.00	0.00	43.60	28.05	28.03	0.00	0.00	8.42	20.91	0.00	0.00	35.43	28.32	25.63	0.00	0.00
S. equorum	182	8.11	27.93	0.00	0.00	42.12	38.05	29.42	0.00	0.00	6.27	20.64	0.00	0.00	38.19	36.91	26.10	0.00	0.00
S. equorum	184	9.52	18.07	0.00	307.97	25.56	23.10	28.66	1.00	7209.69	10.29	24.18	0.00	807.62	36.11	31.26	25.26	8.97	9417.76
S. equorum	187	8.01	23.77	12.29	0.00	37.96	34.70	27.45	0.00	0.00	5.01	23.10	21.30	0.00	35.26	38.56	25.69	0.00	0.00
S. equorum	189	18.73	54.00	7.19	139.90	95.75	18.61	30.04	0.00	8373.88	12.65	25.83	0.00	417.24	43.24	25.53	23.76	3.53	9969.74
S. equorum	190	12.46	30.01	1.50	155.97	45.32	11.91	29.31	0.00	7426.63	12.71	23.94	0.00	711.63	37.47	20.07	25.12	6.34	7813.24
S. equorum	191	16.25	36.43	5.24	166.88	50.52	11.49	28.44	0.00	8251.17	14.04	29.10	0.00	713.43	47.39	19.87	25.51	6.44	8312.85
S. equorum	192	9.87	26.14	2.01	437.39	36.17	31.96	25.53	89.80	80.92	7.40	22.80	0.00	970.32	36.86	34.10	24.22	191.57	163.52
S. equorum	196	8.05	24.98	0.00	0.00	31.40	24.14	26.68	0.00	0.00	7.06	19.08	0.00	0.00	31.70	25.69	23.21	0.00	0.00
S. equorum	197	7.59	28.61	0.00	0.00	40.06	26.93	27.88	0.00	0.00	6.10	16.36	0.00	0.00	24.45	29.44	25.56	0.00	0.00
S. equorum	199	9.81	34.66	2.34	0.00	58.14	31.01	29.50	0.00	0.00	6.80	20.05	0.00	0.00	34.37	29.15	23.92	0.00	0.00
S. equorum	202	7.70	22.69	0.00	0.00	31.89	25.13	26.10	0.00	0.00	6.14	18.04	0.00	0.00	29.56	26.50	24.18	0.00	0.00
S. equorum	205	59.66	27.82	2.81	0.00	38.23	22.43	28.65	0.00	0.00	81.28	16.08	0.00	0.00	24.97	24.93	25.44	0.00	0.00
S. equorum	208	7.72	22.84	0.00	0.00	31.86	23.12	25.81	0.00	0.00	8.38	29.47	0.00	0.00	50.11	28.16	27.31	0.00	0.00
S. equorum	210	8.33	27.52	0.00	0.00	36.52	29.16	28.04	0.00	0.00	9.96	30.98	0.00	0.00	44.26	33.53	28.90	0.00	0.00
S. equorum	211	13.05	46.75	3.36	0.00	77.62	30.97	30.15	0.00	0.00	9.85	22.03	0.00	0.00	38.65	33.49	27.42	0.00	0.00
S. equorum	217	10.57	24.20	0.20	0.00	36.35	25.36	27.37	0.00	0.00	8.20	21.16	0.00	0.00	35.07	26.78	25.46	0.00	0.00
S. equorum	219	10.97	23.25	0.43	0.00	37.97	28.64	26.55	0.00	0.00	9.40	26.88	0.00	0.00	46.10	32.31	25.54	0.00	0.00
S. equorum	221	9.31	23.16	1.46	0.00	38.62	28.93	27.62	0.00	0.00	8.55	25.97	0.00	0.00	45.14	30.83	26.22	0.00	0.00
S. equorum	223	9.01	16.84	0.00	0.00	23.14	28.88	27.09	0.00	0.00	5.02	18.27	0.00	0.00	29.20	36.03	27.48	0.00	0.00
S. equorum	225	9.28	14.15	0.00	0.00	26.96	24.87	26.11	0.00	0.00	6.76	24.59	0.00	0.00	37.21	30.82	26.09	0.00	0.00

S. equorum	226	9.69	13.74	1.28	147.51	24.95	21.13	27.37	0.00	5699.61	7.81	17.53	0.00	303.71	20.78	29.91	25.80	1.36	7821.53
S. equorum	227	8.22	19.35	0.00	0.00	31.31	25.64	26.06	0.00	0.00	9.67	25.49	0.00	0.00	42.07	32.29	26.75	0.00	0.00
S. equorum	231	10.18	21.81	0.00	0.00	38.76	32.49	26.21	0.00	0.00	6.04	17.71	0.00	0.00	32.27	33.46	24.75	0.00	0.00
S. equorum	232	6.96	20.04	0.00	0.00	30.72	28.53	26.79	0.00	0.00	5.40	14.98	0.00	0.00	24.74	31.33	26.34	0.00	0.00
S. equorum	233	10.53	18.77	0.26	303.71	28.37	18.56	25.79	0.60	5896.18	9.39	21.73	0.00	696.81	35.65	27.44	24.66	6.93	9953.28
S. equorum	235	8.71	15.99	0.00	0.00	27.66	25.42	42.03	0.00	0.00	6.65	22.04	0.00	0.00	36.73	29.82	25.81	0.00	0.00
S. equorum	236	7.99	20.34	0.00	0.00	25.09	31.89	37.55	0.00	0.00	5.12	19.88	0.00	0.00	29.11	36.37	25.51	0.00	0.00
S. equorum	239	6.66	15.64	0.00	0.00	20.41	24.62	29.46	0.00	0.00	5.44	16.70	0.00	0.00	27.61	27.41	24.79	0.00	0.00
S. equorum	245	8.45	22.28	0.00	0.00	32.26	19.83	27.18	0.00	0.00	6.30	17.10	0.00	0.00	32.44	22.51	23.87	0.00	0.00
S. equorum	250	7.29	17.15	0.00	0.00	23.77	27.51	27.60	0.00	0.00	9.00	26.98	0.00	0.00	50.21	31.94	26.43	0.00	0.00
S. equorum	252	7.04	15.15	0.00	0.00	20.20	26.96	26.75	0.00	0.00	6.97	21.48	0.00	0.00	37.31	30.41	25.02	0.00	0.00
S. equorum	256	8.49	19.44	0.65	230.96	29.79	15.49	25.76	0.00	6388.21	11.53	25.86	0.00	457.31	47.80	26.57	24.37	2.95	9388.43
S. equorum	257	5.60	11.63	0.00	0.00	19.14	22.05	25.24	0.00	0.00	5.74	11.89	0.00	0.00	18.32	27.80	23.62	0.00	0.00
S. equorum	260	65.55	22.97	1.18	0.00	32.41	20.17	28.00	0.00	0.00	109.99	24.81	0.00	0.00	39.88	23.94	25.16	0.00	0.00
S. equorum	263	9.41	17.58	0.00	0.00	26.12	26.71	26.97	0.00	0.00	5.07	17.39	0.00	0.00	26.77	29.34	24.57	0.00	0.00
S. equorum	265	7.82	11.16	0.00	0.00	18.11	26.72	27.63	0.00	0.00	6.66	18.60	0.00	0.00	30.25	30.39	24.94	0.00	0.00
S. equorum	267	7.90	17.54	0.00	0.00	28.45	24.54	25.11	0.00	0.00	3.78	10.22	0.00	0.00	20.75	27.63	23.50	0.00	0.00
S. equorum	268	12.12	32.86	2.89	0.00	46.38	28.92	29.31	0.00	0.00	8.86	30.02	0.00	0.00	48.93	30.54	26.05	0.00	0.00
S. equorum	270	9.33	23.19	0.00	0.00	37.51	31.00	28.30	0.00	0.00	8.36	25.29	0.00	0.00	43.02	39.41	27.92	0.00	0.00
S. equorum	271	11.72	23.33	0.00	0.00	34.16	25.26	27.56	0.00	0.00	8.05	22.26	0.00	0.00	30.65	32.83	26.07	0.00	0.00
S. equorum	273	8.03	22.81	0.00	0.00	35.40	21.89	27.65	0.00	0.00	6.45	15.02	0.00	0.00	27.93	25.23	24.60	0.00	0.00
S. equorum	274	12.01	26.42	0.00	0.00	36.62	28.80	27.99	0.00	0.00	8.09	23.07	0.00	0.00	33.84	34.96	25.79	0.00	0.00
S. equorum	277	8.48	22.50	0.00	0.00	27.34	24.38	26.86	0.00	0.00	6.91	24.23	0.00	0.00	39.93	28.63	25.58	0.00	0.00
S. equorum	278	14.99	30.54	3.34	178.00	48.91	20.01	28.04	0.00	7061.88	7.68	13.69	0.00	309.54	26.79	29.88	23.90	1.51	8234.05
S. equorum	280	13.91	30.95	0.00	0.00	44.69	36.96	27.91	0.00	0.00	6.28	18.19	0.00	0.00	32.51	34.70	23.95	0.00	0.00
S. equorum	281	9.17	21.26	0.00	0.00	29.69	26.79	26.80	0.00	0.00	6.69	20.59	0.00	0.00	31.54	29.33	25.82	0.00	0.00
S. equorum	282	10.24	21.28	0.00	0.00	31.56	25.43	28.67	0.00	0.00	7.03	19.68	0.00	0.00	29.49	28.85	25.46	0.00	0.00
S. equorum	284	13.83	25.78	2.35	206.72	42.34	14.94	27.12	0.00	7331.58	8.91	13.63	0.00	311.71	23.36	26.96	22.59	1.61	7780.32
S. equorum	295	7.27	20.18	0.00	0.00	32.00	28.97	27.92	0.00	0.00	4.64	19.17	0.00	0.00	33.69	29.57	24.39	0.00	0.00
S. equorum	296	10.15	25.99	0.00	0.00	35.44	28.38	27.99	0.00	0.00	4.14	16.89	0.00	0.00	29.82	27.26	24.80	0.00	0.00

S. equorum	298	8.45	23.79	0.00	0.00	26.77	27.36	27.61	0.00	0.00	5.77	23.02	0.00	0.00	29.53	30.52	24.43	0.00	0.00
S. equorum	300	11.13	29.87	0.27	0.00	44.75	35.88	27.16	0.00	0.00	4.81	12.89	0.00	0.00	27.44	39.85	26.36	0.00	0.00
S. equorum	306	6.77	20.01	0.00	0.00	26.37	25.45	26.32	0.00	0.00	9.79	31.68	0.00	0.00	46.13	35.10	28.47	0.00	0.00
S. equorum	313	7.70	17.93	0.00	0.00	27.09	26.70	26.39	0.00	0.00	5.28	11.60	0.00	0.00	19.85	33.10	54.56	0.00	0.00
S. equorum	314	9.31	18.47	0.00	0.00	28.26	26.45	27.15	0.00	0.00	8.01	27.40	0.00	0.00	41.71	36.05	53.03	0.00	0.00
S. equorum	315	7.96	22.25	0.00	0.00	30.52	26.79	25.64	0.00	0.00	7.11	19.29	0.00	0.00	32.59	36.58	47.29	0.00	0.00
S. equorum	316	11.43	32.16	0.00	0.00	50.18	29.37	28.31	0.00	0.00	6.15	12.73	0.00	0.00	21.61	31.57	37.31	0.00	0.00
S. equorum	317	7.04	14.52	0.00	0.00	18.47	27.16	27.21	0.00	0.00	4.30	11.29	0.00	0.00	21.51	30.29	34.52	0.00	0.00
S. equorum	318	8.27	17.42	0.00	0.00	23.73	26.38	26.67	0.00	0.00	6.19	18.80	0.00	0.00	28.71	32.01	33.61	0.00	0.00
S. equorum	321	11.69	25.88	0.00	0.00	38.70	28.85	28.12	0.00	0.00	7.01	18.30	0.00	0.00	29.19	31.92	31.69	0.00	0.00
S. equorum	322	9.43	21.69	0.00	0.00	30.27	26.72	27.15	0.00	0.00	6.04	13.41	0.00	0.00	22.30	31.22	30.87	0.00	0.00
S. equorum	323	9.23	28.17	2.27	0.00	39.70	27.24	27.75	0.00	0.00	4.87	9.24	0.00	0.00	19.61	27.82	28.49	0.00	0.00
S. equorum	325	9.53	20.89	0.31	0.00	31.01	29.77	28.37	0.00	0.00	3.94	10.01	0.00	0.00	20.32	34.16	30.37	0.00	0.00
S. equorum	327	7.14	18.30	0.00	0.00	19.97	27.04	26.43	0.00	0.00	4.23	12.02	0.00	0.00	18.02	38.50	30.88	0.00	0.00
S. fleuretti	47	7.93	28.85	1.88	0.00	44.57	28.05	30.20	0.00	0.00	10.42	18.94	0.00	0.00	34.48	35.26	29.58	0.00	0.00
S. haemolyticus	49	11.79	32.27	2.43	0.00	42.82	33.89	28.73	0.00	0.00	10.98	23.39	0.00	0.00	34.24	40.92	268.97	0.00	0.00
S. haemolyticus	51	12.09	37.50	3.18	0.00	52.82	36.41	29.17	0.00	0.00	7.44	18.19	0.00	0.00	27.57	39.38	36.03	0.00	0.00
S. hominis	53	9.22	36.48	4.75	0.00	50.69	33.88	29.00	0.00	0.00	9.09	16.55	0.00	0.00	28.25	35.90	28.11	0.00	0.00
S. lugdunensis	92	282.80	44901.89	767.09	0.00	1732.01	31.22	28.36	0.00	0.00	394.37	52105.13	903.26	0.00	1678.58	36.81	27.06	0.00	0.00
S. pasteuri	55	8.72	31.25	1.29	0.00	40.66	31.39	28.46	0.00	0.00	10.11	26.00	0.00	0.00	38.86	32.70	28.11	0.00	0.00
S. pasteuri	56	9.81	30.19	0.00	0.00	39.51	33.69	28.09	0.00	0.00	8.05	19.26	0.00	0.00	30.06	35.92	28.14	0.00	0.00
S. pasteuri	57	14.40	44.59	3.83	0.00	66.23	37.77	30.64	0.00	0.00	7.81	21.84	0.00	0.00	33.81	37.16	28.51	0.00	0.00
S. pasteuri	96	12.54	39.49	3.59	0.00	49.15	28.44	28.22	0.00	0.00	9.40	24.00	0.00	0.00	34.47	30.38	26.48	0.00	0.00
S. pasteuri	222	13.13	31.99	2.60	0.00	47.61	30.12	29.12	0.00	0.00	4.84	18.28	0.00	0.00	20.73	30.06	26.62	0.00	0.00
S. saprophyticus	4	8.50	36.48	1.56	0.00	43.58	24.04	28.59	0.00	0.00	7.34	20.44	0.00	0.00	31.58	22.42	27.16	0.00	0.00
S. saprophyticus	34	8.74	22.48	0.00	0.00	30.62	27.94	27.60	0.00	0.00	7.40	22.80	0.00	0.00	35.76	24.83	25.20	0.00	0.00
S. saprophyticus	37	11.96	38.55	5.66	0.00	57.15	26.32	28.25	0.00	0.00	8.06	20.52	0.00	0.00	36.46	24.42	25.01	0.00	0.00
S. saprophyticus	40	5.93	20.51	0.00	0.00	29.36	22.86	27.21	0.00	0.00	7.81	24.04	0.00	0.00	33.80	22.33	25.76	0.00	0.00
S. saprophyticus	54	8.96	22.85	0.00	0.00	35.49	29.70	25.04	0.00	0.00	8.42	18.82	0.00	0.00	33.16	31.99	26.42	0.00	0.00
S. saprophyticus	58	13.04	50.63	4.69	0.00	82.84	20.23	30.26	0.00	0.00	9.61	21.67	0.00	0.00	32.30	21.18	26.37	0.00	0.00

S. saprophyticus	59	8.08	27.70	0.94	0.00	36.53	15.88	27.30	0.00	0.00	6.78	18.17	0.00	0.00	31.41	14.99	24.53	0.00	0.00
S. saprophyticus	61	7.74	28.61	0.00	0.00	44.86	28.13	26.82	0.00	0.00	8.26	23.61	0.00	0.00	35.36	28.76	25.32	0.00	0.00
S. saprophyticus	62	7.99	23.01	0.00	0.00	33.80	14.30	26.59	0.00	0.00	5.76	21.21	0.00	0.00	32.32	17.74	26.05	0.00	0.00
S. saprophyticus	63	7.12	20.63	0.00	0.00	31.45	14.93	26.72	0.00	0.00	8.53	19.03	0.00	0.00	31.29	14.64	23.25	0.00	0.00
S. saprophyticus	64	7.03	21.71	0.00	0.00	26.90	13.05	25.43	0.00	0.00	6.85	24.04	0.00	0.00	35.96	16.63	25.24	0.00	0.00
S. saprophyticus	65	10.12	27.97	1.23	0.00	38.41	19.84	26.76	0.00	0.00	8.71	21.75	0.00	0.00	35.89	20.07	25.75	0.00	0.00
S. saprophyticus	73	8.23	27.02	0.00	0.00	38.10	18.30	27.19	0.00	0.00	6.78	20.38	0.00	0.00	36.15	23.00	26.45	0.00	0.00
S. saprophyticus	91	11.91	41.67	2.44	0.00	59.70	17.73	26.68	0.00	0.00	4.03	5.46	0.00	0.00	12.89	21.02	24.51	0.00	0.00
S. saprophyticus	114	8.84	33.03	0.00	0.00	50.55	25.89	27.39	0.00	0.00	7.12	21.58	0.00	0.00	33.12	22.10	25.41	0.00	0.00
S. saprophyticus	115	10.05	362.22	2.10	0.00	44.91	35.73	28.56	0.00	0.00	5.85	390.88	0.00	0.00	27.42	35.04	24.66	0.00	0.00
S. saprophyticus	117	95.67	24.40	0.00	0.00	16.43	25.44	54.22	0.00	0.00	119.01	2.25	0.00	0.00	0.00	21.92	22.24	0.00	0.00
S. saprophyticus	157	7.69	24.03	0.00	0.00	36.64	33.71	28.82	0.00	0.00	10.39	32.47	0.00	0.00	56.95	43.81	32.67	0.00	0.00
S. saprophyticus	185	12.62	35.57	0.00	0.00	49.67	28.56	28.78	0.00	0.00	4.55	15.97	0.00	0.00	21.92	24.63	24.60	0.00	0.00
S. saprophyticus	188	59.54	26.78	1.08	0.00	21.91	25.30	24.09	0.00	0.00	106.23	6.75	0.00	0.00	0.00	23.69	21.80	0.00	0.00
S. saprophyticus	220	11.95	32.36	0.00	0.00	48.54	18.71	25.32	0.00	0.00	4.37	18.77	0.00	0.00	27.43	21.30	24.78	0.00	0.00
S. saprophyticus	230	9.79	20.25	0.00	0.00	32.28	24.11	25.21	0.00	0.00	11.82	31.11	0.00	0.00	49.10	26.36	24.65	0.00	0.00
S. saprophyticus	234	8.62	21.05	0.00	0.00	18.51	36.11	65.12	0.00	0.00	0.00	7.29	0.00	0.00	0.00	33.10	22.57	0.00	0.00
S. saprophyticus	241	8.52	19.67	0.00	0.00	23.52	16.80	26.04	0.00	0.00	9.74	32.78	0.00	0.00	43.20	18.94	25.50	0.00	0.00
S. saprophyticus	243	7.57	14.91	0.00	0.00	21.87	15.52	26.50	0.00	0.00	9.24	30.59	0.00	0.00	56.52	17.62	26.24	0.00	0.00
S. saprophyticus	247	17.55	43.54	0.00	0.00	73.18	36.12	33.28	0.00	0.00	8.49	25.64	0.00	0.00	36.38	35.11	25.90	0.00	0.00
S. saprophyticus	276	9.97	24.20	2.07	154.10	30.23	11.30	25.96	0.00	6983.45	7.80	18.93	0.00	283.97	15.97	20.86	22.55	1.11	8578.95
S. saprophyticus	285	13.32	34.55	0.34	0.00	50.15	19.03	26.84	0.00	0.00	9.33	28.80	0.00	0.00	46.84	21.18	24.38	0.00	0.00
S. saprophyticus	286	9.40	16.85	0.00	0.00	23.90	25.59	26.61	0.00	0.00	7.37	20.91	0.00	0.00	28.68	28.11	24.34	0.00	0.00
S. saprophyticus	304	11.56	26.43	0.00	0.00	37.76	33.81	28.64	0.00	0.00	8.95	26.44	0.00	0.00	47.02	42.63	29.46	0.00	0.00
S. saprophyticus	311	7.94	17.85	0.00	0.00	26.94	16.65	25.81	0.00	0.00	9.54	18.63	0.00	0.00	33.73	32.91	116.98	0.00	0.00
S. saprophyticus	328	9.42	25.08	0.00	0.00	38.61	23.01	27.99	0.00	0.00	7.94	17.78	0.00	0.00	28.42	26.81	29.88	0.00	0.00
S. saprophyticus	329	9.42	16.29	0.00	0.00	23.26	15.46	25.27	0.00	0.00	6.35	17.08	0.00	0.00	24.99	18.61	26.95	0.00	0.00
S. sciuri	70	9.54	28.04	0.34	0.00	41.11	31.65	27.84	0.00	0.00	5.09	16.67	0.00	0.00	26.56	37.18	26.55	0.00	0.00
S. sciuri	72	7.47	25.74	0.00	0.00	35.16	33.73	27.87	0.00	0.00	6.84	18.92	0.00	0.00	31.92	42.74	29.05	0.00	0.00
S. sciuri	144	8.88	33.56	2.41	0.00	57.71	30.41	29.53	0.00	0.00	5.54	15.71	0.00	0.00	29.85	46.46	29.73	0.00	0.00

S. simulans	66	6.22	26.48	0.00	0.00	31.57	34.20	27.22	0.00	0.00	7.47	28.45	0.00	0.00	36.62	40.06	25.72	0.00	0.00
S. simulans	95	11.87	50.35	2.42	0.00	56.45	39.41	29.62	0.00	0.00	8.23	30.70	0.00	0.00	33.39	44.40	27.50	0.00	0.00
S. simulans	229	6.63	16.03	0.00	0.00	20.96	23.18	25.55	0.00	0.00	7.10	21.34	0.00	0.00	38.90	26.58	24.75	0.00	0.00
S. succinus	67	10.54	29.45	2.91	0.00	42.99	27.54	28.84	0.00	0.00	6.94	19.68	0.00	0.00	30.70	29.93	26.65	0.00	0.00
S. succinus	68	6.83	20.68	0.00	0.00	27.54	24.90	26.83	0.00	0.00	6.05	14.37	0.00	0.00	25.26	26.27	26.78	0.00	0.00
S. succinus	69	8.21	31.25	4.10	0.00	43.05	24.99	29.00	0.00	0.00	10.07	21.48	0.00	0.00	35.54	23.39	26.13	0.00	0.00
S. succinus	89	9.09	30.96	1.16	0.00	34.68	26.48	27.20	0.00	0.00	6.40	18.55	0.00	0.00	34.97	28.11	25.55	0.00	0.00
S. vitulinus	261	15.65	23.64	1.99	169.21	41.98	19.72	27.94	0.00	6848.97	9.55	21.25	0.00	314.48	36.12	28.53	24.88	2.06	9104.06
S. warneri	74	7.64	19.39	0.00	0.00	27.90	34.78	28.88	0.00	0.00	9.92	23.36	0.00	0.00	40.47	38.65	27.95	0.00	0.00
S. warneri	90	9.07	31.10	0.94	0.00	41.09	29.54	27.45	0.00	0.00	9.47	19.53	0.00	0.00	36.12	29.97	26.37	0.00	0.00
S. warneri	112	9.45	32.91	1.97	0.00	44.34	31.56	26.92	0.00	0.00	9.55	22.09	0.00	0.00	39.72	29.97	26.27	0.00	0.00
S. warneri	113	7.76	26.03	1.38	0.00	35.88	28.15	26.96	0.00	0.00	7.84	17.67	0.00	0.00	31.77	29.14	25.72	0.00	0.00
S. xylosus	76	8.26	28.79	0.61	0.00	43.20	28.60	26.01	0.00	0.00	7.65	18.70	0.00	0.00	33.80	31.30	23.66	0.00	0.00
S. xylosus	77	5.88	26.22	1.55	0.00	38.98	31.93	26.20	0.00	0.00	5.76	13.39	0.00	0.00	26.60	32.99	24.78	0.00	0.00
S. xylosus	78	9.64	32.19	2.71	0.00	48.85	31.37	26.37	0.00	0.00	6.40	9.97	0.00	0.00	18.76	31.34	24.05	0.00	0.00
S. xylosus	79	10.48	29.01	1.12	0.00	39.49	30.57	26.73	0.00	0.00	7.14	14.94	0.00	0.00	28.12	30.25	22.84	0.00	0.00
S. xylosus	80	9.03	28.42	0.00	0.00	43.09	29.28	25.76	0.00	0.00	5.93	10.60	0.00	0.00	24.14	30.37	22.60	0.00	0.00
S. xylosus	81	8.22	26.67	1.43	0.00	40.63	23.91	24.43	0.00	0.00	3.00	8.79	0.00	0.00	15.35	24.76	22.61	0.00	0.00
S. xylosus	82	5.54	18.12	0.00	0.00	25.79	23.27	24.01	0.00	0.00	5.31	13.66	0.00	0.00	26.71	24.59	22.59	0.00	0.00
S. xylosus	83	12.03	39.50	6.22	0.00	54.55	34.23	27.00	0.00	0.00	7.13	13.50	0.00	0.00	24.65	33.19	24.98	0.00	0.00
S. xylosus	84	10.07	31.98	4.49	0.00	46.96	28.33	24.13	0.00	0.00	3.96	13.29	0.00	0.00	22.18	29.31	22.53	0.00	0.00
S. xylosus	85	9.46	25.34	0.00	0.00	31.43	26.45	24.92	0.00	0.00	7.52	13.82	0.00	0.00	21.90	30.17	23.12	0.00	0.00
S. xylosus	86	10.01	33.84	1.64	0.00	44.02	29.16	25.92	0.00	0.00	3.75	10.79	0.00	0.00	22.86	28.77	22.04	0.00	0.00
S. xylosus	87	6.33	28.62	0.62	0.00	38.89	26.56	24.84	0.00	0.00	7.16	12.06	0.00	0.00	30.28	25.99	23.26	0.00	0.00
S. xylosus	88	8.18	23.22	0.00	0.00	30.82	26.45	26.03	0.00	0.00	7.76	12.93	0.00	0.00	20.61	28.11	23.19	0.00	0.00
S. xylosus	102	4.68	19.68	0.00	0.00	30.40	25.73	24.37	0.00	0.00	7.49	13.85	0.00	0.00	23.23	27.91	23.94	0.00	0.00
S. xylosus	103	9.38	28.61	1.09	0.00	34.41	27.94	25.66	0.00	0.00	6.81	14.18	0.00	0.00	23.50	28.29	22.60	0.00	0.00
S. xylosus	104	9.50	31.25	0.35	0.00	48.65	28.40	25.24	0.00	0.00	8.41	13.29	0.00	0.00	24.59	29.14	22.84	0.00	0.00
S. xylosus	105	9.88	28.73	1.80	0.00	37.69	30.60	26.44	0.00	0.00	7.34	11.71	0.00	0.00	23.41	29.14	22.19	0.00	0.00
S. xylosus	106	7.49	21.38	0.00	0.00	27.60	26.41	23.48	0.00	0.00	7.28	18.73	0.00	0.00	33.60	28.87	22.97	0.00	0.00

S. xylosus	109	10.15	35.18	2.67	0.00	47.82	21.04	25.85	0.00	0.00	5.67	19.91	0.00	0.00	30.33	24.53	25.43	0.00	0.00
S. xylosus	111	9.98	32.98	1.04	0.00	45.52	36.12	29.82	0.00	0.00	8.06	23.39	0.00	0.00	36.45	36.11	25.77	0.00	0.00
S. xylosus	125	15.75	42.35	8.35	204.80	113.14	11.70	35.61	0.10	17044.02	10.31	16.16	0.00	649.85	25.88	20.51	43.50	4.97	6908.38
S. xylosus	128	198.39	1971.56	20.77	408.11	17126.80	14.55	24.68	0.00	0.00	7.05	1937.27	24.52	554.09	27873.75	16.64	32.50	0.00	0.00
S. xylosus	335	8.04	19.44	0.00	0.00	23.45	25.30	24.15	0.00	0.00	6.80	11.44	0.00	0.00	15.22	28.11	26.44	0.00	0.00
S. xylosus	121	0.00	0.00	0.00	0.00	0.00	8.32	18.19	0.00	0.00	3.83	100.28	0.00	0.00	23.90	35.07	23.86	0.00	0.00
S. xylosus	123	74.37	28.13	0.38	0.00	37.69	19.46	28.96	0.00	0.00	161.13	21.28	3.07	0.00	38.44	40.70	96.23	0.00	0.00
S. xylosus	129	6.63	18.41	0.00	0.00	46.27	25.72	24.13	0.00	0.00	7.43	25.01	0.00	0.00	368.56	27.96	29.30	0.00	0.00
S. xylosus	130	12.33	35.73	2.09	0.00	55.08	29.12	26.96	0.00	0.00	5.78	11.47	0.00	0.00	44.44	28.76	28.04	0.00	0.00
S. xylosus	132	14.42	40.77	3.81	166.82	51.77	27.00	27.15	0.00	7809.67	10.42	12.78	0.00	400.37	21.49	27.78	25.60	2.76	7884.06
S. xylosus	134	12.01	42.25	6.61	0.00	64.26	29.69	25.65	0.00	0.00	7.22	16.89	0.00	0.00	24.68	30.23	24.91	0.00	0.00
S. xylosus	135	8.35	25.56	0.00	0.00	37.70	27.95	25.61	0.00	0.00	6.66	14.51	0.00	0.00	28.12	29.44	25.00	0.00	0.00
S. xylosus	137	12.15	44.68	1.73	0.00	67.55	30.83	27.99	0.00	0.00	5.06	15.50	0.00	0.00	24.59	29.61	25.12	0.00	0.00
S. xylosus	145	12.71	50.98	5.68	0.00	76.22	30.73	31.86	0.00	0.00	6.95	16.25	0.00	0.00	31.00	31.09	28.10	0.00	0.00
S. xylosus	152	77.16	39.81	6.28	0.00	58.44	22.87	32.32	0.00	0.00	101.37	23.25	0.00	0.00	32.21	24.24	25.09	0.00	0.00
S. xylosus	154	10.95	20.80	2.27	0.00	27.57	11.85	22.34	0.00	0.00	4.18	4.30	0.00	0.00	0.00	18.25	22.15	0.00	0.00
S. xylosus	155	68.71	43.63	7.02	0.00	68.36	22.74	28.32	0.00	0.00	87.04	14.95	0.00	0.00	25.04	24.53	24.49	0.00	0.00
S. xylosus	158	8.39	24.58	1.42	0.00	34.99	29.39	25.98	0.00	0.00	2.35	7.11	0.00	0.00	13.09	29.07	23.97	0.00	0.00
S. xylosus	159	7.94	29.61	0.00	0.00	38.08	29.00	24.72	0.00	0.00	7.97	17.01	0.00	0.00	27.81	29.65	22.91	0.00	0.00
S. xylosus	161	8.20	24.85	0.00	0.00	38.84	29.22	26.02	0.00	0.00	5.15	16.25	0.00	0.00	26.59	31.07	24.41	0.00	0.00
S. xylosus	162	8.58	17.89	0.00	0.00	27.89	29.59	26.95	0.00	0.00	6.13	20.27	0.00	0.00	32.96	30.09	23.12	0.00	0.00
S. xylosus	165	12.62	35.08	5.79	0.00	59.54	31.83	28.16	0.00	0.00	5.03	9.76	0.00	0.00	17.97	34.67	26.11	0.00	0.00
S. xylosus	167	8.42	30.00	0.43	0.00	37.56	29.15	27.17	0.00	0.00	7.15	16.67	0.00	0.00	32.65	29.89	24.24	0.00	0.00
S. xylosus	170	7.75	28.48	0.00	0.00	36.14	28.02	25.48	0.00	0.00	8.97	18.77	0.00	0.00	31.84	30.08	23.56	0.00	0.00
S. xylosus	174	9.78	24.24	0.72	0.00	34.93	28.33	25.18	0.00	0.00	7.13	14.43	0.00	0.00	23.18	30.19	22.63	0.00	0.00
S. xylosus	177	8.02	21.30	0.00	0.00	37.05	26.91	28.86	0.00	0.00	7.03	23.94	0.00	0.00	38.41	24.04	26.63	0.00	0.00
S. xylosus	179	9.28	24.12	0.63	167.65	31.26	22.72	28.52	0.00	7371.61	12.78	30.60	0.00	365.84	52.85	31.03	26.47	2.09	10010.21
S. xylosus	180	8.35	28.69	0.00	0.00	42.57	35.40	30.74	0.00	0.00	5.47	24.37	0.00	0.00	30.14	36.81	26.00	0.00	0.00
S. xylosus	193	8.81	29.26	0.00	0.00	46.18	26.61	25.46	0.00	0.00	5.77	19.78	0.00	0.00	33.78	28.19	23.71	0.00	0.00
S. xylosus	194	7.08	19.22	0.00	0.00	28.42	26.02	24.77	0.00	0.00	7.86	20.48	0.00	0.00	33.87	28.31	22.48	0.00	0.00

S. xylosus	195	10.72	27.60	0.00	0.00	38.12	26.82	25.00	0.00	0.00	7.98	15.52	0.00	0.00	26.58	26.88	22.36	0.00	0.00
S. xylosus	198	12.32	43.75	1.38	0.00	65.20	29.94	26.31	0.00	0.00	6.36	14.90	0.00	0.00	23.38	28.28	22.78	0.00	0.00
S. xylosus	200	62.62	30.42	0.08	0.00	43.42	23.31	29.59	0.00	0.00	93.61	21.82	0.00	0.00	41.25	24.23	24.43	0.00	0.00
S. xylosus	201	8.67	21.67	0.43	0.00	34.46	25.45	26.30	0.00	0.00	10.96	24.54	0.00	0.00	41.50	29.12	23.80	0.00	0.00
S. xylosus	203	11.33	38.49	3.61	0.00	56.75	29.44	26.72	0.00	0.00	7.74	20.67	0.00	0.00	28.49	27.88	22.61	0.00	0.00
S. xylosus	204	7.08	23.10	0.00	0.00	30.20	25.21	26.43	0.00	0.00	7.78	29.24	0.00	0.00	44.15	29.75	27.57	0.00	0.00
S. xylosus	206	5.57	23.38	0.00	0.00	32.35	8.80	24.20	0.00	0.00	7.68	23.59	0.00	0.00	43.05	13.65	22.99	0.00	0.00
S. xylosus	207	10.76	27.88	1.25	0.00	39.81	26.52	24.74	0.00	0.00	5.94	12.91	0.00	0.00	23.61	26.26	24.37	0.00	0.00
S. xylosus	209	8.23	23.86	0.00	0.00	33.62	26.31	25.61	0.00	0.00	7.98	17.59	0.00	0.00	27.01	28.85	23.74	0.00	0.00
S. xylosus	212	8.70	28.74	0.00	0.00	36.70	30.36	28.79	0.00	0.00	6.88	16.83	0.00	0.00	26.04	29.47	24.18	0.00	0.00
S. xylosus	213	11.25	30.31	0.57	0.00	47.43	28.65	26.22	0.00	0.00	6.93	21.65	0.00	0.00	32.26	30.15	23.63	0.00	0.00
S. xylosus	214	9.91	19.99	0.00	0.00	31.09	25.04	25.63	0.00	0.00	7.53	23.37	0.00	0.00	37.02	29.36	25.75	0.00	0.00
S. xylosus	215	13.17	27.68	3.91	0.00	45.45	27.13	25.56	0.00	0.00	7.46	18.85	0.00	0.00	33.38	29.82	23.63	0.00	0.00
S. xylosus	216	8.26	22.25	0.00	0.00	33.53	27.02	25.32	0.00	0.00	9.60	29.16	0.00	0.00	51.11	31.28	25.06	0.00	0.00
S. xylosus	218	8.53	16.51	0.00	0.00	27.61	25.21	23.90	0.00	0.00	5.90	19.95	0.00	0.00	32.11	28.25	23.39	0.00	0.00
S. xylosus	224	69.56	28.78	4.58	0.00	42.59	22.27	28.87	0.00	0.00	82.54	18.74	0.00	0.00	34.08	26.25	25.56	0.00	0.00
S. xylosus	228	12.70	28.15	0.00	0.00	44.69	32.86	28.88	0.00	0.00	8.84	22.76	0.00	0.00	38.92	36.51	28.90	0.00	0.00
S. xylosus	238	9.36	20.08	0.07	0.00	27.39	27.14	29.91	0.00	0.00	11.44	26.21	0.00	0.00	49.79	34.63	27.22	0.00	0.00
S. xylosus	242	7.68	20.80	0.00	0.00	31.06	26.20	27.16	0.00	0.00	7.06	15.50	0.00	0.00	28.46	27.78	24.35	0.00	0.00
S. xylosus	244	8.16	20.00	0.00	0.00	31.84	25.44	26.20	0.00	0.00	6.38	13.68	0.00	0.00	21.71	27.15	23.69	0.00	0.00
S. xylosus	246	8.11	17.19	0.00	0.00	26.72	24.87	24.37	0.00	0.00	6.10	14.41	0.00	0.00	21.94	28.17	24.39	0.00	0.00
S. xylosus	249	10.86	24.90	0.00	0.00	34.77	27.38	26.40	0.00	0.00	8.00	18.47	0.00	0.00	30.52	30.13	24.00	0.00	0.00
S. xylosus	251	10.06	25.89	0.00	0.00	39.38	27.77	26.44	0.00	0.00	5.40	11.49	0.00	0.00	18.44	28.71	23.29	0.00	0.00
S. xylosus	253	8.62	21.67	0.00	0.00	30.27	26.48	25.58	0.00	0.00	7.34	15.73	0.00	0.00	26.17	29.90	23.25	0.00	0.00
S. xylosus	255	5.88	20.80	0.00	0.00	24.24	25.85	24.62	0.00	0.00	3.53	6.57	0.00	0.00	12.57	28.88	22.41	0.00	0.00
S. xylosus	258	13.28	29.54	0.00	0.00	48.46	28.56	26.78	0.00	0.00	3.10	13.33	0.00	0.00	21.03	27.15	21.57	0.00	0.00
S. xylosus	259	7.49	19.02	0.00	0.00	27.22	26.46	25.70	0.00	0.00	9.16	17.04	0.00	0.00	25.58	28.70	22.39	0.00	0.00
S. xylosus	262	7.11	14.83	0.00	0.00	24.13	25.35	25.10	0.00	0.00	3.88	11.74	0.00	0.00	22.20	27.66	22.59	0.00	0.00
S. xylosus	264	11.26	26.33	0.00	0.00	38.60	35.19	29.14	0.00	0.00	7.51	20.50	0.00	0.00	31.56	33.86	26.29	0.00	0.00
S. xylosus	266	4.53	11.81	0.00	0.00	15.30	25.33	24.57	0.00	0.00	6.06	16.70	0.00	0.00	24.64	29.46	23.14	0.00	0.00

S. xylosus	269	9.13	22.64	0.00	0.00	31.65	27.81	25.89	0.00	0.00	4.19	7.30	0.00	0.00	18.29	30.15	23.48	0.00	0.00
S. xylosus	272	11.31	29.33	0.56	0.00	39.99	29.59	26.34	0.00	0.00	8.14	18.01	0.00	0.00	26.19	29.81	23.09	0.00	0.00
S. xylosus	275	11.03	25.69	0.00	0.00	33.26	27.21	25.52	0.00	0.00	5.48	10.55	0.00	0.00	14.99	30.51	23.33	0.00	0.00
S. xylosus	279	9.03	17.41	0.00	0.00	24.19	24.37	23.33	0.00	0.00	5.12	13.21	0.00	0.00	17.67	28.28	22.79	0.00	0.00
S. xylosus	283	7.63	21.79	0.00	0.00	27.90	26.31	24.94	0.00	0.00	8.75	21.70	0.00	0.00	28.01	30.56	23.41	0.00	0.00
S. xylosus	287	8.69	20.93	0.00	0.00	30.69	26.85	24.91	0.00	0.00	5.35	10.75	0.00	0.00	20.98	28.16	22.44	0.00	0.00
S. xylosus	289	7.27	16.03	0.00	0.00	21.75	23.99	24.78	0.00	0.00	4.35	8.53	0.00	0.00	13.33	26.92	24.45	0.00	0.00
S. xylosus	290	3.94	5.77	0.00	0.00	3.10	24.33	23.45	0.00	0.00	7.84	22.60	0.00	0.00	35.55	29.01	22.44	0.00	0.00
S. xylosus	292	9.64	24.45	0.00	0.00	33.56	27.10	25.39	0.00	0.00	5.35	12.99	0.00	0.00	22.26	29.65	23.70	0.00	0.00
S. xylosus	294	9.69	21.34	0.00	0.00	32.05	26.17	25.44	0.00	0.00	7.49	13.13	0.00	0.00	20.58	31.21	23.95	0.00	0.00
S. xylosus	297	12.45	25.22	0.00	0.00	39.61	28.06	27.03	0.00	0.00	5.48	14.42	0.00	0.00	20.28	28.64	22.48	0.00	0.00
S. xylosus	301	8.64	20.42	0.00	0.00	32.69	28.00	25.97	0.00	0.00	7.56	16.15	0.00	0.00	25.93	31.95	24.14	0.00	0.00
S. xylosus	307	8.48	19.87	0.16	0.00	29.85	25.03	25.17	0.00	0.00	6.48	21.81	0.00	0.00	31.99	26.68	23.05	0.00	0.00
S. xylosus	310	7.99	24.84	0.00	0.00	34.91	27.12	25.37	0.00	0.00	6.89	16.93	0.00	0.00	27.97	30.03	23.77	0.00	0.00
S. xylosus	312	5.69	12.36	0.00	0.00	14.54	25.26	24.87	0.00	0.00	9.50	17.60	0.00	0.00	27.28	38.20	93.61	0.00	0.00
S. xylosus	319	7.47	18.20	0.00	0.00	27.79	27.02	24.78	0.00	0.00	8.22	11.91	0.00	0.00	20.55	31.98	31.97	0.00	0.00
S. xylosus	320	7.31	15.42	0.00	0.00	25.55	25.02	23.64	0.00	0.00	10.69	23.69	0.00	0.00	43.80	32.57	32.76	0.00	0.00
S. xylosus	326	7.70	20.76	0.00	0.00	27.72	25.21	24.38	0.00	0.00	5.56	14.75	0.00	0.00	25.30	29.00	27.12	0.00	0.00
Staphylococcus sp.	22	7.67	27.87	0.14	0.00	31.01	40.61	30.05	0.00	0.00	4.62	21.53	0.00	0.00	35.01	44.65	30.42	0.00	0.00
Staphylococcus sp.	23	8.13	33.53	2.09	0.00	41.93	33.90	30.46	0.00	0.00	5.67	16.89	0.00	0.00	29.25	41.42	29.81	0.00	0.00
Staphylococcus sp.	24	9.28	27.62	0.18	0.00	38.84	40.08	30.81	0.00	0.00	8.01	22.23	0.00	0.00	37.96	42.99	30.96	0.00	0.00

© 2019 by the authors. Submitted for possible open access publication under the terms and conditions of the Creative Commons Attribution (CC BY) license (http://creativecommons.org/licenses/by/4.0/).
